# Psychological Aspects and Mental Health Risks in Children and Adolescents with Congenital Heart Defects—A Systematic Review

**DOI:** 10.3390/diagnostics16091271

**Published:** 2026-04-23

**Authors:** Cristina Tecar, Lacramioara Eliza Chiperi, Bianca-Elena Iftimie, Livia Livint-Popa, Maria Balea, Silvina Ilut, Nicu Catalin Draghici, Dafin Fior Muresanu

**Affiliations:** 1 RoNeuro Institute for Neurological Research and Diagnostic, 400364 Cluj-Napoca, Romania; cristina_pantelemon@yahoo.com (C.T.); livia.popa@umfcluj.ro (L.L.-P.); maria.balea@ssnn.ro (M.B.); silvina.ilut@yahoo.com (S.I.); nicu.draghici@umfcluj.ro (N.C.D.); dafin.muresanu@umfcluj.ro (D.F.M.); 2 Monza Ares Hospital, 400347 Cluj-Napoca, Romania; 3 Department of Mother and Child, Iuliu Hațieganu University of Medicine and Pharmacy, 400347 Cluj-Napoca, Romania; 4 Department of Neurosciences, Psychiatry and Pediatric Psychiatry, Iuliu Hațieganu University of Medicine and Pharmacy, 400347 Cluj-Napoca, Romania; biancaiftimie4@gmail.com; 5 Department of Neuroscience, Iuliu Hațieganu University of Medicine and Pharmacy, 400083 Cluj-Napoca, Romania

**Keywords:** congenital heart defect, mental health, children, quality of life, psychological outcomes, neurodevelopment, Attention-Deficit Hyperactivity Disorder (ADHD), autism spectrum disorder, anxiety, depression

## Abstract

**Background/Objectives:** Congenital heart defects (CHDs) are the most common congenital anomalies, and survival into adolescence and adulthood now exceeds 90%. Increasing evidence suggests that children and adolescents with CHD face elevated risks of psychological, psychiatric and neurodevelopmental disorders. This systematic review aims to synthesize recent evidence on mental health outcomes, cognitive profiles, quality of life and associated risk factors in pediatric CHD. **Methods:** This review was conducted according to PRISMA 2020 guidelines. Five databases (PubMed/MEDLINE, Embase, Scopus, Web of Science, Cochrane Library) were searched for studies published between January 2015 and November 2025. Eligible studies (observational, interventional and neuroimaging) included participants aged 0–18 years with any type of CHD and reported psychological, psychiatric, neurodevelopmental, cognitive or health-related quality-of-life outcomes. Due to substantial heterogeneity, findings were synthesized narratively. **Results:** Sixty-one studies involving over 120,000 participants were included. Children and adolescents with CHD showed increased prevalence of anxiety, depression, attention-deficit/hyperactivity disorder, autism spectrum disorder and post-traumatic stress symptoms compared with peers without CHD. Neurodevelopmental impairments, particularly in executive functioning, attention and memory, were frequently reported, especially in complex CHD and single-ventricle physiology. Health-related quality of life was consistently reduced, mainly in emotional and social domains. Parental mental health, disease severity and cumulative medical burden were significant correlates. Neuroimaging studies identified structural and functional brain alterations associated with cognitive and emotional vulnerability. **Conclusions:** Pediatric CHD is associated with substantial psychological and neurodevelopmental burden, particularly in complex disease. Early identification and integration of routine psychological care within multidisciplinary CHD programs are essential to improve long-term outcomes.

## 1. Introduction

Congenital heart defects (CHDs) are the most prevalent congenital disorders, affecting 1% of live births with varying degrees of severity, from mild (not requiring emergency surgery or likely to require surgery) to severe and complex (requiring surgery in the first months or years of life) [1]. For a long time, CHD were considered specific to the pediatric population, but with the progress made in recent years in their treatment, the survival rate of these children has increased significantly over time, with >90% of them reaching adulthood [2].

While long-term outcomes in adults with CHD have been increasingly studied [3,4,5,6], including psychological and psychosocial functioning, less is known about how these difficulties emerge and evolve during childhood and adolescence. It is important that children with CHD receive psychological monitoring before reaching adulthood so that they can receive appropriate support from medical and mental health professionals [7].

A number of risk factors for adverse psychological outcomes have been identified that exert their influence as early as in the intrauterine period. These may include: impaired fetal circulation, hypoxia, brain dysmaturation, cardiac arrest, perioperative neurological impairment, use of cardiopulmonary mechanical support, medication, prolonged hospitalization, exposure to stress, a number of genetic and epigenetic factors, and parental mental health [8,9]. It is known that innate and environmental factors, such as genetic factors, underlying the common developmental pathways of the brain and heart, are known to be more important risk factors than perioperative factors [2].

Importantly, psychological outcomes in children with congenital heart defects must be interpreted within a developmental framework, as the type and expression of these outcomes vary substantially across different age groups.

The pediatric population with complex CHD or those with associated comorbidities are at higher risk for autism spectrum disorder (ASD), attention-deficit/hyperactivity disorder (ADHD), neurodevelopmental disorder, or developmental delay with subsequent functional consequences [8,10,11]. School-aged children, who underwent surgery for CHD in their first year of life, are more likely to develop symptoms of ADHD [11]. Collaboration between the pediatric cardiologist and the school counselor is important in order to improve educational, psychological, and social outcomes [2]. It has been observed that attention-deficit is more common in patients with cyanotic heart defects and in those with single-ventricle defects [12]. In a meta-analysis conducted by Marshall KH et al., poorer emotional functioning in children with single-ventricle CHD was associated with older age at Fontan operation, while poorer social functioning was associated with the presence of hypoplastic left-sided heart syndrome [13]. Children and adolescents with CHD often undergo painful medical procedures and prolonged hospitalization. They often need multiple invasive procedures from an early age and require medical visits throughout their lives, which can lead to acute stress reactions or even long-term post-traumatic stress disorder (PTSD) [14]. In adolescents with CHD, depression and loneliness are closely linked and have been associated with a lower quality of life [15].

Despite the growing body of literature on psychological and neurodevelopmental outcomes in children with congenital heart defects, existing studies are highly heterogeneous in terms of populations, methodologies, and outcome measures. Moreover, many studies focus on specific conditions, age groups, or isolated outcomes, without providing an integrated overview of mental health risks across the pediatric CHD spectrum [2,16].

To date, there is a lack of a comprehensive and up-to-date synthesis that simultaneously addresses psychiatric, neurodevelopmental, cognitive, and quality-of-life outcomes, together with their associated risk factors.

Therefore, this systematic review aims to provide a structured and integrative synthesis of recent evidence on psychological and mental health outcomes in children and adolescents with CHD, with a focus on identifying consistent patterns, risk factors, and gaps in current knowledge.

## 2. Methods

### 2.1. Study Design

This study was conducted as a systematic review following the Preferred Reporting Items for Systematic Reviews and Meta-Analyses (PRISMA 2020) guidelines [17]. The PRISMA checklist is provided in Supplementary File S1. The objective was to synthesize evidence on psychological outcomes, mental health risks, and neurodevelopmental profiles in children and adolescents with CHD.

A review protocol was developed prior to study selection. Although it was not registered in a public database, the methodology was defined a priori and strictly followed throughout the review process, with no post hoc modifications.

### 2.2. Search Strategy and Data Source

A comprehensive literature search was conducted across multiple electronic databases, including PubMed/MEDLINE, Scopus, Embase, Cochrane Library and Web of Science Core Collection (Clarivate Analytics), from January 2015 to November 2025. The time restriction (2015–2025) was applied to ensure inclusion of contemporary evidence reflecting advances in surgical management, survival rates, and evolving understanding of neurodevelopmental and psychological outcomes in CHD populations.

The search strategy combined Medical Subject Headings (MeSH) and free-text terms related to: (1) congenital heart disease; (2) pediatric population; (3) psychological, psychiatric, neurodevelopmental, cognitive, or quality-of-life outcomes, using Boolean operators (AND, OR).

The search was limited to studies published in English. Reference lists of included studies were also screened to identify additional relevant articles. The review was conducted in accordance with PRISMA 2020 guidelines.

Syntax was adapted to each database. The full search strategies for each database are provided in Supplementary File S2.

### 2.3. Eligibility Criteria

#### 2.3.1. Inclusion Criteria

Studies were included if they met the following criteria: Population: children and adolescents aged 0–18 years with any CHD (simple, moderate, or complex; cyanotic or acyanotic; single- or biventricular physiology); Study design: observational studies (cohort, case–control, cross-sectional); randomized or non-randomized interventional studies; neuroimaging investigations examining psychological, cognitive, or emotional outcomes; population-based registry studies; Outcomes: at least one psychological, psychiatric, behavioral, neurodevelopmental, cognitive, or quality-of-life outcome (e.g., anxiety, depression, PTSD, ADHD, ASD, executive function, memory, behavior problems); Language: only studies published in English were considered due to feasibility constraints; Availability: full-text accessible.

#### 2.3.2. Exclusion Criteria

We excluded: (1) studies involving adults (>18 years) or mixed samples without extractable pediatric data; (2) articles focusing only on perioperative neurological complications without psychological outcomes; (3) case reports, reviews, editorials, conference abstracts; (4) studies evaluating exclusively cardiac or surgical outcomes without mental health measures; (5) studies focusing only on genetic syndromes (e.g., Trisomy 21, 22q11.2 deletion) without specifying CHD-related psychological findings.

#### 2.3.3. Studies Grouping for Synthesis

For the narrative synthesis, the included studies were grouped according to their primary psychological or neurodevelopmental outcomes. Given the substantial clinical and methodological heterogeneity across study designs, CHD severity, age ranges, and assessment instruments, we organized the evidence into thematic domains reflecting the main constructs examined across the literature.

Studies were therefore synthesized within the following predefined categories: (1) psychiatric disorders (ADHD, ASD, anxiety, depression, PTSD); (2) emotional and behavioral problems; (3) cognitive and executive functioning; (4) neurodevelopmental outcomes; (5) health-related quality of life and psychosocial functioning; (6) parental and family psychological factors; (7) neuroimaging correlates of psychological or cognitive outcomes; and (8) interventional or preventive psychological programs. This grouping allowed for a structured comparison of findings within conceptually similar domains despite the differences in study methodologies.

### 2.4. Study Selection

**Figure 1:**
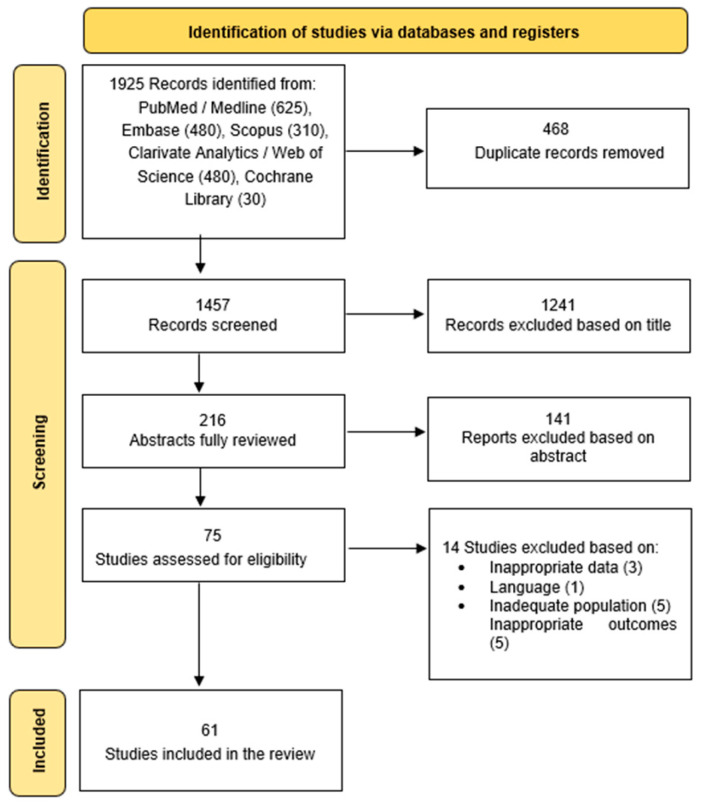
PRISMA flow diagram for new systematic review: selection process of included studies.

All records retrieved from the database searches were imported into a reference management system. Duplicate records were removed automatically and manually. Two independent reviewers screened titles and abstracts in a non-blinded mode according to the inclusion criteria. Full-text review was performed for studies that appeared eligible. Disagreements were resolved through discussion or consultation with a third reviewer. The study selection process is illustrated in Figure 1 (PRISMA flow diagram). A total of 61 studies met the eligibility criteria and were included in the qualitative synthesis.

### 2.5. Data Extraction

Data were extracted independently by two reviewers using a standardized extraction form. The following information was collected from each study: (1) authors, year, country, and study design; (2) sample size and participant characteristics (age, sex, CHD type and severity); (3) psychological, psychiatric, cognitive or neurodevelopmental outcomes; (4) psychometric instruments or neuroimaging methods used; (5) parental or family psychological variables (when applicable); (6) main statistical results and effect estimates; (7) identified predictors, moderators, or mediators of outcomes. Discrepancies in extracted data were resolved through consensus.

When studies provided incomplete or unclear information, we applied several standardized assumptions to ensure consistency across the synthesis. If the age range was not explicitly reported, but the study population was described as “children,” “adolescents,” or “pediatric patients,” we assumed an age range of 0–18 years, unless otherwise specified. When CHD severity (simple, moderate, complex) was not clearly defined, we used the authors’ categorization as presented, without reclassification. If psychological or neurodevelopmental assessment tools were mentioned without detailed scoring methods, we assumed the instruments were administered and interpreted according to their standard validated protocols. For studies reporting effect estimates without confidence intervals or exact *p*-values, we interpreted the findings qualitatively based on the authors’ reported significance. No imputation was performed for missing numerical data and outcomes were synthesized using the available information only.

### 2.6. Risk of Bias Assessment

Because no meta-analysis was conducted, formal statistical assessments of reporting bias (such as funnel plots or Egger’s tests) were not applicable. The methodological quality and risk of bias of the included studies were assessed using standardized tools appropriate to each study design.

For randomized controlled trials, the Cochrane Risk of Bias tool version 2 (RoB 2.0) was applied, evaluating domains including randomization process, deviations from intended interventions, missing outcome data, measurement of outcomes, and selection of reported results.

For non-randomized interventional and observational studies (cohort, case–control, and cross-sectional designs), the Risk Of Bias In Non-randomized Studies of Interventions (ROBINS-I) tool was used. This tool assesses bias due to confounding, participant selection, classification of interventions/exposures, deviations from intended interventions, missing data, measurement of outcomes, and selection of reported results.

The overall risk of bias for each study was categorized as low, moderate, serious, or critical (for ROBINS-I), and as low risk, some concerns, or high risk (for RoB 2.0). The results of the assessment can be found in Supplementary File S5 and are summarized visually using a bar chart.

### 2.7. Effect Measures

Given the heterogeneity of study designs and outcome measures, several effect measures were used to synthesize and present results across outcomes. For dichotomous psychiatric and neurodevelopmental outcomes (e.g., ADHD, ASD, anxiety, depression, PTSD), studies typically reported risk ratios (RRs), odds ratios (ORs), or hazard ratios (HRs); these measures were extracted and presented narratively. For continuous psychological or cognitive outcomes (e.g., executive function, IQ scores, memory performance, behavioral scales, quality-of-life domains), we extracted mean scores, mean differences (MDs) and standardized mean differences (SMDs) when applicable. Correlational outcomes, such as associations between psychological symptoms and clinical parameters, were summarized using correlation coefficients (r) or regression coefficients, (β) as reported by the authors. Because of substantial methodological variability, effect measures were not pooled statistically; instead, they were presented descriptively within each thematic domain.

### 2.8. Data Synthesis

#### 2.8.1. Data Preparation Methods

Minimal data preparation was required due to the heterogeneity of the included literature. When summary statistics were missing (e.g., standard deviations not reported), we relied on the qualitative interpretation provided by the study authors, rather than on imputing values. No mathematical conversions or transformations were applied to incomplete data. When studies reported outcomes using different scoring directions or subscale structures, results were presented using the original formats to preserve comparability and avoid incorrect harmonization.

#### 2.8.2. Tabulation and Visual Presentation of Results

Results of individual studies were organized into structured tables summarizing key characteristics (sample size, CHD type, age, outcomes measured, instruments used and main findings). Tables were grouped according to the synthesis domains to allow straightforward comparison across similar outcomes. Narrative summaries accompanied each table.

#### 2.8.3. Methods for Synthesizing Results

Because of significant clinical, methodological and measurement heterogeneity among the included studies—including wide variability in psychological instruments, CHD classifications, age ranges and study designs—a narrative synthesis approach was used. Findings were synthesized qualitatively within predefined thematic domains (psychiatric, emotional/behavioral, cognitive, neurodevelopmental, quality of life, family factors, neuroimaging, and interventional outcomes.

A meta-analysis was considered; however, it was deemed inappropriate due to substantial heterogeneity in study populations (age ranges, CHD types), outcome measures (neurodevelopmental, emotional, behavioral), and study designs. The variability in assessment tools and reporting formats further limited the comparability of effect sizes across studies.

#### 2.8.4. Exploration of Heterogeneity

Given the narrative nature of the synthesis, no formal statistical exploration of heterogeneity (e.g., subgroup analysis, meta-regression) was performed. However, potential sources of heterogeneity—such as CHD severity, single—versus biventricular physiology, age at assessment, type of psychological instrument used and presence of associated comorbidities—were qualitatively examined and highlighted within each thematic domain.

#### 2.8.5. Sensitivity Analyses

Due to the heterogeneity of study designs, populations, and outcome measures, a quantitative meta-analysis was not feasible. Consequently, formal statistical sensitivity analyses (e.g., leave-one-out analysis) could not be performed.

Instead, a narrative sensitivity analysis was conducted by comparing findings across studies with varying levels of methodological quality, as assessed by the risk of bias tools (RoB 2.0 and ROBINS-I). Particular attention was given to whether studies at lower risk of bias reported consistent results compared to those with moderate or serious risk of bias, in order to evaluate the robustness of the overall conclusions.

## 3. Results

### 3.1. Study Selection and General Characteristics

A total of 61 studies met the inclusion criteria [7,10,16,18,19,20,21,22,23,24,25,26,27,28,29,30,31,32,33,34,35,36,37,38,39,40,41,42,43,44,45,46,47,48,49,50,51,52,53,54,55,56,57,58,59,60,61,62,63,64,65,66,67,68,69,70,71,72,73,74,75] and were synthesized in the present review (Figure 1). The included studies were published between 2015 and 2025 and originated from Europe, North America, Asia, and Australia, reflecting a broad geographic distribution. The studies evaluated over 120,000 children and adolescents with CHD across diverse healthcare settings. Study designs comprised population-based cohort studies, cross-sectional observational studies, case–control studies, longitudinal follow-up studies, randomized controlled trials, interventional pilot studies and neuroimaging investigations, including structural MRI, functional MRI, EEG and machine-learning-based trajectory analyses.

Sample sizes varied widely, ranging from small clinically referred cohorts (e.g., neuroimaging studies with <50 participants [49,51]) to large nationwide population-based samples, including tens of thousands of participants [17,22,27]. Studies included children and adolescents with CHD, with ages spanning from infancy to young adulthood. Several studies incorporated healthy control groups, siblings, or population controls, while others relied on normative test references.

The spectrum of CHD severity was broad and included simple defects (e.g., ventricular septal defect, atrial septal defect), moderate lesions (e.g., tetralogy of Fallot, transposition of the great arteries) and complex or critical CHD, particularly single-ventricle physiology and Fontan circulation. Multiple studies stratified outcomes according to CHD complexity, surgical history, or age at repair [34,36]. Characteristics of included studies are synthetized in Supplementary File S3.

During the full-text screening stage, studies were excluded for several reasons [76,77,78,79,80,81,82,83,84,85,86,87,88,89]. The most common reasons for exclusion included: (1) ineligible population (e.g., studies not focusing on children or adolescents with congenital heart defects), (2) outcomes not related to psychological, neurodevelopmental, or psychosocial functioning, (3) inappropriate study design (e.g., case reports, reviews, editorials), and (4) insufficient or unavailable data. A summary of the main reasons for exclusion is provided in the PRISMA flow diagram and Supplementary File S4.

**Figure 2:**
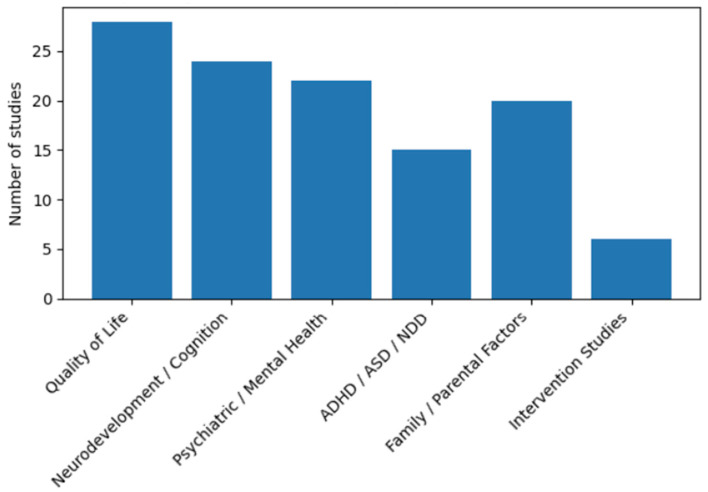
Distribution of outcomes across included studies. The bar chart illustrates the number of studies addressing each outcome domain within the included literature. Quality of life, neurodevelopmental/cognitive outcomes and psychiatric or emotional health outcomes are most frequently reported, whereas intervention-based studies remain markedly underrepresented.

Figure 2 illustrates the distribution of outcome domains across the included studies. The majority of studies focused on quality of life, neurodevelopmental and cognitive outcomes and psychiatric or emotional health, reflecting a strong descriptive emphasis on the burden and risk characterization in children and adolescents with congenital heart disease. Disorders such as anxiety, depression, attention-deficit/hyperactivity disorder, autism spectrum disorder and broader neurodevelopmental disorders were frequently examined, often in relation to disease severity, surgical complexity or neurobiological correlates.

Family- and parent-related psychological factors were also commonly assessed, underscoring the growing recognition of the bidirectional relationship between child outcomes and caregiver mental health. In contrast, intervention-based studies constituted only a small proportion of the available evidence, with relatively few randomized controlled trials or structured psychological programs targeting cognitive, emotional or resilience-related outcomes [24,31,57].

For the purpose of this review, psychological outcomes were conceptually grouped into four distinct domains: (1) psychiatric outcomes (including clinically diagnosed disorders and symptom-based measures such as anxiety, depression, and behavioral disorders), (2) neurodevelopmental outcomes (including global developmental delay, motor development, and early developmental milestones), (3) cognitive outcomes (including intelligence, executive functioning, attention, and academic performance), and (4) psychosocial and quality-of-life outcomes (including social functioning, emotional well-being, family impact, and health-related quality of life).

These domains were analyzed separately to ensure conceptual clarity, while also acknowledging potential overlap between them in clinical contexts. Across studies, several consistent patterns emerge. Neurodevelopmental and cognitive vulnerabilities appear most strongly associated with medical complexity, while psychiatric and psychosocial outcomes demonstrate greater variability and are influenced by both medical and environmental factors.

### 3.2. Psychiatric Outcomes

Psychiatric outcomes (including clinically diagnosed disorders and symptom-based measures such as anxiety, depression, and behavioral disorders), were assessed in the majority of the included studies. Elevated rates of internalizing symptoms, including anxiety and depressive symptoms, were consistently reported across age groups, particularly among adolescents with moderate-to-severe CHD [33,56]. These outcomes were more commonly reported in school-aged children and adolescents, where emotional and behavioral symptoms become more clinically apparent.

Population-based cohort studies demonstrated a significantly increased risk of diagnosed psychiatric disorders, including mood disorders, anxiety disorders and stress-related conditions, compared with the general population. For example, Tsao et al. [22] reported hazard ratios between 1.5 and 2.0 for ADHD and ASD, while Miles et al. [16] observed an increased risk of psychiatric diagnoses (HR ~1.6) in large national cohorts. Similarly, Khanna et al. [52] reported an overall increased prevalence of mental illness across the lifespan in CHD populations. Children and adolescents with complex CHD or single-ventricle physiology exhibited the highest burden of psychiatric morbidity.

Several studies specifically examined post-traumatic stress symptoms, both in patients and parents, identifying clinically relevant levels of traumatic stress associated with early surgeries, intensive care exposure and ongoing medical surveillance. For instance, Konkel et al. [18] reported that approximately 20–25% of children and adolescents with CHD screened positive for traumatic stress symptoms. Emotional vulnerability and reduced psychological well-being were particularly evident in adolescents with Fontan circulation.

### 3.3. Neurodevelopmental Outcomes

Neurodevelopmental outcomes (including global developmental delay, motor development, and early developmental milestones) were evaluated in a substantial subset of studies, using standardized developmental tests and neuropsychological batteries. These outcomes were primarily assessed in infancy and early childhood, reflecting the developmental relevance of early milestone acquisition. These impairments were more frequently reported in children with complex or cyanotic CHD. Population-based and registry data demonstrated an increased risk of neurodevelopmental disorders, with odds ratios ranging from 1.3 to 1.6 in Liu et al.’s study [20] and relative risks up to 1.8 for developmental disabilities in Delgado et al.’s study [27].

Executive function deficits, attention impairments, and memory difficulties were among the most frequently reported cognitive abnormalities. Large effect sizes for executive dysfunction were described in adolescents with Fontan physiology [43], while structural MRI studies demonstrated reduced hippocampal volumes associated with memory impairment [30].

Attention and executive functioning deficits were among the most frequently identified cognitive impairments, particularly in cohorts with transposition of the great arteries, tetralogy of Fallot, Fontan circulation and neonatal arch repair [38,43,44].

Neuroimaging studies [49,51] revealed structural and functional brain alterations, including reduced volumes in the hippocampus, caudate nuclei and regions involved in emotion regulation, memory and executive functioning. Functional connectivity and EEG studies further supported atypical neural network organization, underlying working memory and attentional control deficits [40].

### 3.4. Cognitive Outcomes

Cognitive outcomes including intelligence, executive functioning, attention, and academic performance were analyzed in a subset of studies [34,47,54,55], using educational performance indicators.

Longitudinal cohort studies showed that these neurodevelopmental vulnerabilities persist into school age and adolescence, with significant implications for academic performance and increased need for special education services [47,54]. Early surgical timing and disease severity were identified as important predictors of outcomes [34,55].

### 3.5. Quality of Life and Psychosocial Functioning

Health-related quality of life (HRQoL) was assessed in more than half of the included studies using validated child- and parent-reported instruments. Overall, children and adolescents with CHD reported lower HRQoL, compared with healthy peers, particularly in the emotional, social and school functioning domains [7,28,45].

Reduced HRQoL was more pronounced in patients with greater disease complexity, exercise intolerance, repeated hospitalizations and ongoing medical treatment [42,53]. Some studies highlighted discrepancies between child self-reports and parent proxy reports, with parents often perceiving poorer quality of life than the children themselves [26].

Interventional and pilot studies suggested that resilience-building programs, structured exercise interventions and psychological integration within multidisciplinary cardiac care may be associated with improvements in psychosocial functioning and HRQoL, although evidence remains limited and heterogeneous [24,31,57].

**Figure 3:**
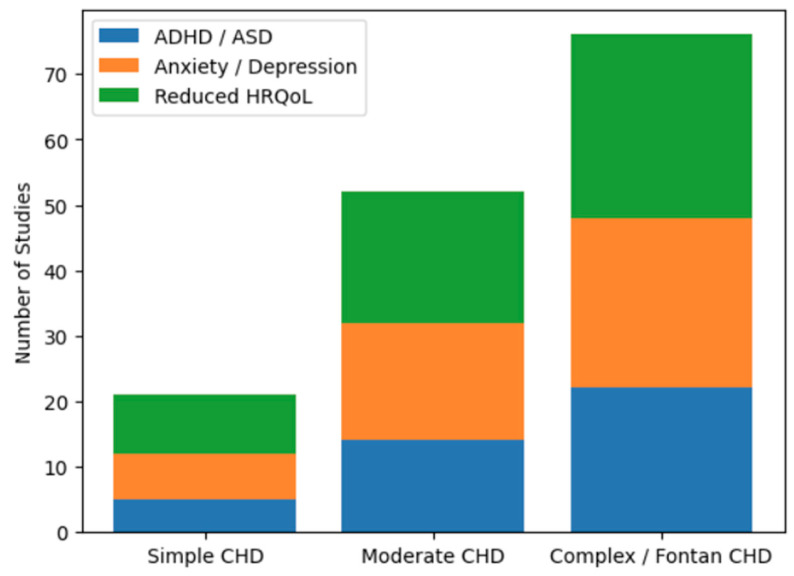
Relationship between CHD severity and psychological, neurodevelopmental burden, health-related quality of life.

Severity-based comparisons were performed using frequency-based synthesis, categorizing studies according to the predominant CHD severity reported and the psychological or neurodevelopmental outcomes assessed. Figure 3 illustrates the distribution of psychological and neurodevelopmental outcomes across studies stratified by CHD severity. The stacked bars represent the number of studies reporting ADHD or ASD, anxiety or depressive symptoms and reduced HRQoL in children and adolescents with simple, moderate and complex (including Fontan circulation) CHD. Studies involving complex CHD consistently reported a higher psychological and neurodevelopmental burden, compared with those focusing on simple or moderate defects.

Parental and family-related psychological variables were explicitly examined in a subset of studies. Parental mental health, including anxiety, depression and post-traumatic stress symptoms, was shown to be closely associated with child psychological outcomes and HRQoL [37,50]. Parental distress frequently acted as a moderator of intervention effects and as a predictor of child emotional and behavioral problems.

Structural equation modeling and cohort studies demonstrated that parental emotional well-being strongly predicts child quality of life [19], while parenting stress and family socioeconomic status were associated with developmental trajectories and behavioral outcomes [31,46].

### 3.6. Developmental Considerations Across Age Groups

The included studies encompassed a wide range of developmental stages, from infancy to adolescence, with important differences in the types of outcomes assessed at each stage.

In infancy and early childhood, research predominantly focused on neurodevelopmental outcomes, including motor development, language acquisition, and global developmental milestones [21,25]. At these stages, standardized developmental assessments were commonly used, and early delays were frequently identified, particularly in children with complex CHD.

In contrast, studies involving school-aged children more often assessed cognitive functioning and academic performance, including intelligence, executive functioning, and learning abilities as well as emerging neurodevelopmental disorders such as ASD and ADHD [10,20,22]. These findings suggest that early neurodevelopmental vulnerabilities may translate into later cognitive and educational challenges.

Among adolescents, the focus shifted toward psychiatric symptoms and psychosocial outcomes, including anxiety, depression, social functioning, and health-related quality of life [18,19,23]. Self-reported measures were more frequently used in this age group, highlighting subjective experiences of illness and social integration.

Overall, these findings indicate that psychological and neurodevelopmental outcomes in CHD are dynamic and evolve across developmental stages, with early neurodevelopmental impairments potentially contributing to later cognitive and psychosocial difficulties.

### 3.7. Clinical Heterogeneity Across CHD Populations

The included studies encompassed a broad spectrum of congenital heart defects, ranging from simple lesions to complex conditions requiring multiple surgical interventions. This clinical heterogeneity represents an important factor influencing psychological and neurodevelopmental outcomes.

Several studies suggested that children with more complex CHD, including cyanotic lesions or those requiring early and repeated surgical procedures, were at higher risk for neurodevelopmental impairments and cognitive deficits [21,22]. These patients are more likely to experience perioperative complications, prolonged hospitalizations, and altered cerebral perfusion, which may contribute to adverse neurodevelopmental trajectories.

In contrast, studies including children with less complex CHD often reported milder or more variable outcomes, particularly in cognitive and psychosocial domains [20]. However, even in these populations, subtle difficulties in attention, executive functioning, or emotional adjustment were described.

Psychiatric and psychosocial outcomes appeared less consistently associated with lesion complexity. While some studies reported higher levels of anxiety and reduced quality of life in patients with more severe disease [18,19], others suggested that psychosocial outcomes are also strongly influenced by non-medical factors, such as family environment, parental stress, and coping mechanisms [24].

Overall, the findings indicate that neurodevelopmental and cognitive outcomes are more strongly associated with clinical severity and medical burden, whereas psychiatric and psychosocial outcomes appear to be influenced by a more complex interplay of medical and environmental factors.

### 3.8. Neuroimaging Correlates of Psychological and Cognitive Outcomes

A subset of the included studies employed neuroimaging and neurophysiological techniques to investigate the neural correlates of psychological, psychiatric and cognitive outcomes in children and adolescents with congenital heart disease [49,51]. Structural magnetic resonance imaging (MRI), functional MRI, diffusion-based analyses, sleep electroencephalography (EEG) and network-based connectivity approaches were used across cohorts with moderate to complex CHD, most frequently in patients with single-ventricle physiology, Fontan circulation and repaired transposition of the great arteries.

Structural MRI studies consistently reported reduced volumes in brain regions implicated in memory, attention and emotional regulation, including the hippocampus, caudate nuclei and prefrontal and limbic structures [30,51]. These volumetric alterations were significantly associated with memory deficits, attentional problems, increased anxiety symptoms and depressive features. Functional and connectivity-based analyses further demonstrated atypical organization of neural networks, particularly within circuits supporting executive functioning and working memory.

Neurophysiological investigations using sleep EEG revealed altered functional networks underlying working memory abilities, with network inefficiencies correlating with poorer cognitive performance in children with complex CHD [29,40]. Machine-learning approaches applied to multimodal neurodevelopmental data identified distinct neurodevelopmental trajectories, enabling the classification of patients at higher risk for adverse cognitive and behavioral outcomes.

**Figure 4:**
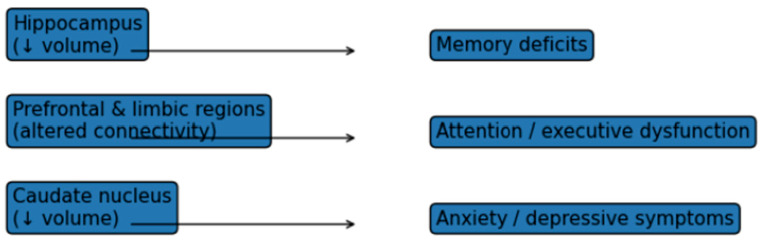
Neuroimaging correlates of psychological and cognitive outcomes in pediatric CHD. ↓ meaning low.

Figure 4 presents a schematic overview of neuroimaging findings reported in children and adolescents with CHD. Structural and functional neuroimaging studies identified reduced hippocampal and caudate nucleus volumes, as well as altered connectivity within prefrontal and limbic networks. These brain alterations were consistently associated with memory deficits, executive and attentional dysfunction and increased anxiety or depressive symptoms.

Collectively, neuroimaging and neurophysiological findings provided objective evidence linking brain structure and network organization to observed psychological and cognitive vulnerabilities in pediatric CHD populations.

### 3.9. Interventional and Preventive Psychological Programs

Studies evaluating psychological and behavioral interventions in children and adolescents with CHD suggest potential benefits in improving emotional well-being, coping strategies, and quality of life. However, most of these studies were based on relatively small sample sizes, pilot designs, or exploratory frameworks [18,22].

Studies included working memory training programs, structured exercise interventions, resilience-focused educational programs and multidisciplinary models integrating psychological care into routine cardiology follow-up. Interventions were delivered in individual, group-based or hybrid formats and varied in duration and intensity.

Working memory interventions demonstrated selective improvements in trained cognitive domains, with variable generalization to broader academic or psychosocial outcomes [57]. Exercise-based interventions were associated with modest improvements in health-related quality of life, with parental mental health identified as a significant moderator of intervention efficacy [31]. Resilience-building programs reported improvements in emotional well-being, coping strategies and perceived psychosocial functioning, although sample sizes were generally small [24].

Preventive approaches emphasizing early identification of psychological risk, routine mental health screening and family-centered psychosocial support were described primarily in observational and pilot studies. Integrated multidisciplinary clinics incorporating psychology services reported improved recognition of emotional and behavioral difficulties and facilitated timely referral for targeted interventions.

**Figure 5:**
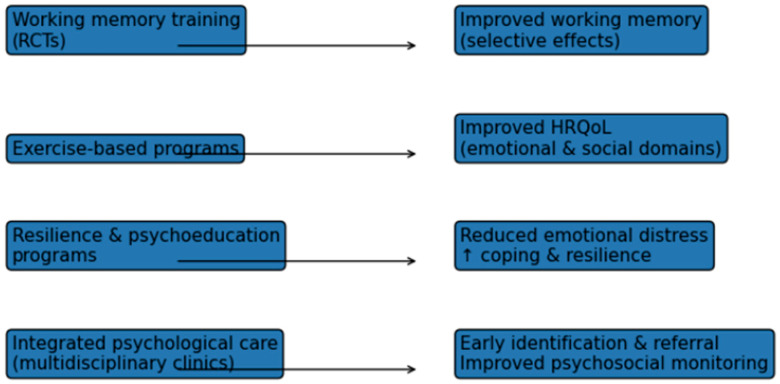
Overview of interventional and preventive psychological approaches in pediatric congenital heart disease. ↑ means increased.

Figure 5 provides an overview of interventional and preventive psychological approaches evaluated in children and adolescents with CHD.

Overall, the evidence base for psychological interventions in pediatric CHD remains heterogeneous, with promising, but preliminary, findings supporting the feasibility and potential benefits of targeted and preventive psychosocial programs.

### 3.10. Identified Predictors, Moderators and Mediators

Across studies, several predictors and moderators of adverse psychological and neurodevelopmental outcomes were consistently identified. These included CHD severity, single-ventricle physiology, younger age at surgery, cumulative surgical exposure, early-term birth, neurodevelopmental vulnerability, reduced exercise capacity and family psychosocial stress [38,43,44].

Protective factors reported in studies included higher resilience, supportive parenting behaviors, better family functioning and access to integrated psychosocial care. In selected analyses, parental mental health and resilience functioned as mediators linking disease-related factors to child outcomes [31].

### 3.11. Summary of Key Findings

Collectively, the included studies demonstrate that children and adolescents with congenital heart disease face a substantially increased risk of psychological distress, psychiatric disorders, neurodevelopmental impairments and reduced quality of life, with the greatest burden observed in those with complex CHD. Outcomes are influenced by a combination of biological, medical and psychosocial factors, underscoring the multifactorial nature of long-term morbidity in this population.

Given the breadth and heterogeneity of the included studies, a summary table (Table 1) was developed to synthesize the main outcomes, together with representative studies and their clinical implications. This structured overview enhances the interpretability of the findings and supports their translation into clinical practice by highlighting consistent patterns and areas requiring targeted intervention.

**Figure 6:**
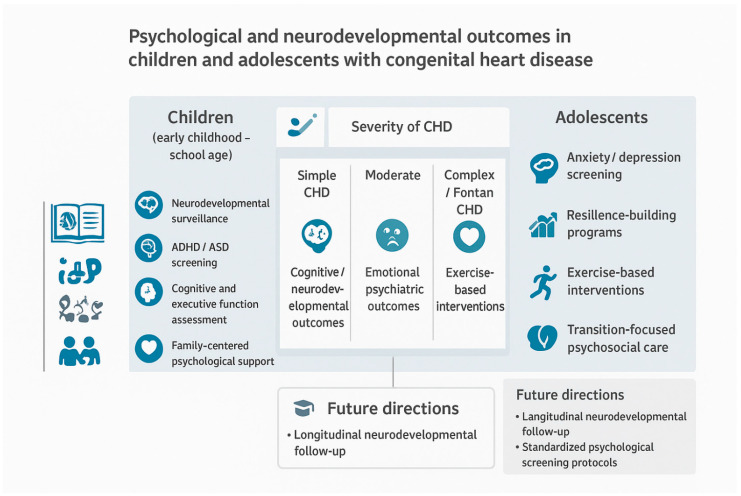
Conceptual framework of psychological and neurodevelopmental outcomes in children and adolescents with congenital heart defects.

Figure 6 summarizes the multidimensional psychological and neurodevelopmental burden associated with congenital heart disease across developmental stages and disease severity. It presents a conceptual framework developed by the authors based on the synthesis of findings across included studies. The framework integrates cognitive, emotional and quality-of-life outcomes, while highlighting age-specific intervention strategies and the current level of supporting evidence. In early childhood and school-age populations, the emphasis is placed on neurodevelopmental surveillance, early identification of attention-deficit/hyperactivity disorder and autism spectrum disorder, structured assessment of executive and cognitive functions and family-centered psychological support. Evidence in this age group is predominantly observational, with a limited number of pilot intervention studies addressing targeted cognitive and behavioral outcomes.

In adolescents with CHD, psychological vulnerability shifts toward internalizing symptoms, including anxiety and depression, reduced resilience and challenges related to autonomy and social integration. Interventions in this population increasingly focus on resilience-building programs, exercise-based psychological interventions and transition-oriented psychosocial care. Compared with younger children, adolescents benefit from a growing body of interventional research, including randomized controlled trials evaluating physical activity-based and psychosocial interventions.

Across the all-age groups, disease complexity remains a major determinant of adverse outcomes, with children and adolescents with complex CHD or Fontan physiology demonstrating the highest burden of cognitive impairment, emotional distress and reduced quality of life. The figure also underscores key gaps in the literature, particularly the scarcity of longitudinal studies tracking psychological trajectories across development and the lack of standardized screening protocols integrated into routine cardiology follow-up.

Finally, the future directions highlighted in Figure 6 advocate for a paradigm shift toward preventive, developmentally tailored psychological care embedded within multidisciplinary CHD programs. Such an approach may facilitate earlier identification of the at-risk individuals, optimize intervention timing and ultimately improve long-term psychosocial and neurodevelopmental outcomes in this growing population.

### 3.12. Investigation of Heterogeneity

Substantial heterogeneity was observed across the included studies with respect to reported psychological, psychiatric and neurodevelopmental outcomes. As summarized in Supplementary File S3 and illustrated in Figure 2, variability in outcomes was associated with differences in disease characteristics, developmental stage, study design and outcome assessment methods. Studies including children with complex congenital heart disease, single-ventricle physiology, or Fontan circulation consistently reported higher prevalence and greater severity of cognitive impairment, emotional symptoms and reduced quality of life, compared with studies involving predominantly simple or moderate defects [38,43,44].

Age at assessment contributed to heterogeneity across outcome domains. Studies focusing on infants and young children more frequently reported neurodevelopmental delay, cognitive impairment and early behavioral difficulties, whereas studies involving adolescents predominantly assessed internalizing symptoms, including anxiety, depression and reduced psychosocial functioning. This age-related variation is reflected in the unequal distribution of outcome domains across studies shown in Figure 2.

Methodological differences further contributed to heterogeneity. The methodological quality of the included studies varied considerably, with differences in sample size, study design, and follow-up duration. Population-based registry studies [17,22,27] generally reported lower prevalence estimates of psychiatric and neurodevelopmental disorders than clinic-based or referral cohorts. Outcome estimates also varied according to assessment modality, with studies using structured diagnostic interviews or standardized neuropsychological testing reporting higher symptom prevalence and larger effect estimates, compared with questionnaire-based assessments. Differences in study design, including cross-sectional versus longitudinal approaches, limited direct comparability across studies.

The inclusion of contextual and family-related variables was another source of heterogeneity. Studies accounting for parental mental health, parenting stress, family functioning or socioeconomic status demonstrated stronger associations with adverse child outcomes than studies that did not include these factors [19,31,37,46,50]. Geographic variation was also evident, with studies conducted in low- and middle-income settings reporting higher symptom burden and lower quality-of-life scores, compared with studies from high-income countries.

Among intervention studies [24,31,57], heterogeneity was observed in intervention type, duration, target age group and selected outcomes, as well as in the sample size. As shown in Figure 2, intervention-based studies represented a small proportion of the overall literature, precluding meaningful comparison across intervention modalities.

A notable source of heterogeneity across the included studies is the variation in outcome measurement. Psychological outcomes were assessed using a wide range of instruments, including standardized neuropsychological tests, parent-report questionnaires, clinician-rated scales, and self-reported measures. These tools capture different dimensions of functioning and are not directly equivalent. For example, objective cognitive test scores reflect cognitive performance, whereas parent-reported measures often capture observable behaviors, and self-reports provide insight into subjective emotional experiences. This variability limits direct comparability across studies and should be considered when interpreting the findings.

Another important consideration is the variability in informant sources. Some studies relied primarily on parent-reported data, while others used adolescent self-report or clinician-administered assessments. These perspectives capture different aspects of psychological functioning. Parent reports tend to reflect observable behaviors and daily functioning, whereas self-reports provide insight into internal emotional states such as anxiety or depressive symptoms. This variability may contribute to differences in reported outcomes across studies.

Overall, heterogeneity across study results reflected variability in disease severity, developmental stage, methodological approach, assessment tools and contextual factors, as documented in Supplementary File S3.

### 3.13. Risk of Bias of Included Studies, Sensitivity Analysis and Certainty of Evidence

Additional analyses were conducted by examining the consistency of findings after excluding studies with limited sample sizes or less clearly defined methodologies. The exclusion of studies with small sample sizes, critical risk of bias, or non-standardized outcome measures did not substantially alter the overall patterns of psychological, psychiatric and neurodevelopmental outcomes across the age groups and disease severity, indicating the consistency of the main findings [76,77,78,79,80,81,82,83,84,85,86,87,88,89]. The overall patterns remained consistent.

The methodological quality of the included studies was variable across designs.

Among the randomized controlled trials, most studies were judged as having “some concerns”, primarily due to limitations in blinding procedures and incomplete reporting of allocation concealment. No studies were rated as having a high overall risk of bias.

For observational studies, the majority were assessed as having a moderate risk of bias, mainly due to potential confounding, selection bias, and heterogeneity in outcome measurement tools. Several large population-based cohort studies demonstrated lower risk of bias, benefiting from robust design and comprehensive registry data. However, smaller single-center and cross-sectional studies were more frequently rated as having serious risk of bias, particularly due to limited sample sizes, lack of control groups, and incomplete adjustment for confounders.

Overall, the evidence base is characterized by a predominance of observational studies with moderate methodological limitations.

**Figure 7:**
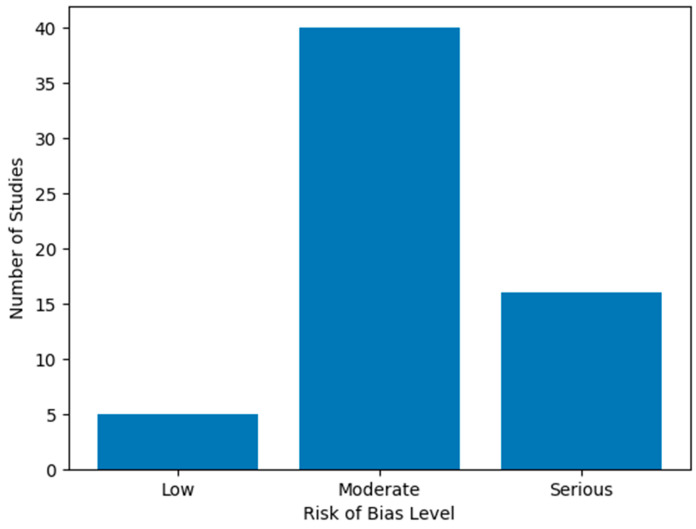
Distribution of risk of bias assessments across included studies.

The distribution of risk of bias assessments across studies is illustrated in Figure 7.

The narrative sensitivity analysis indicated that the main findings were generally consistent across studies with different levels of methodological quality. However, studies with higher risk of bias more frequently reported heterogeneous or less robust associations, which may influence the strength of the conclusions.

The overall certainty of the evidence was assessed using a simplified GRADE approach. Most outcomes were rated as having low to moderate certainty, primarily due to the predominance of observational studies and the presence of moderate to serious risk of bias. Additional downgrading factors included heterogeneity in study populations and outcome measures (inconsistency) and, in some cases, limited sample sizes (imprecision).

No upgrading factors (e.g., large effect size or dose–response gradient) were consistently identified across studies.

## 4. Discussion

### 4.1. Interpretation of the Results in the Context of Other Evidence

Children with chronic illnesses are at increased risk for emotional, behavioral and psychiatric disorders caused by repeated hospitalizations and physical limitations caused by the condition itself [33]. In the present study, we aim to summarize psychological aspects and mental health risks in children and adolescents with congenital heart defects. The summary of findings presented in Table 1 further emphasizes the need for early screening and integrated psychological care in pediatric CHD populations.

An important aspect highlighted by this review is the developmental variability of psychological outcomes in children and adolescents with CHD. The type of outcomes assessed differs significantly across age groups, reflecting both developmental processes and methodological constraints. Early neurodevelopmental impairments identified in infancy may represent the initial manifestation of vulnerability, which can later evolve into cognitive deficits, academic difficulties, and psychosocial challenges during school age and adolescence. This developmental perspective underscores the importance of age-specific screening strategies and longitudinal follow-up in this population.

Another important finding of this review is the impact of clinical heterogeneity on psychological outcomes in children and adolescents with CHD. While neurodevelopmental and cognitive impairments appear to be more strongly associated with disease complexity and surgical burden, psychiatric and psychosocial outcomes are influenced by a broader range of factors, including family environment and individual coping mechanisms. This distinction is clinically relevant, as it suggests that risk stratification should not rely solely on anatomical severity, but should also consider psychosocial and environmental factors when planning follow-up and interventions.

### 4.2. Psychiatric Outcomes

Multiple studies have shown that children with congenital heart defects are at increased risk for neurodevelopmental disorders such as attention-deficit/hyperactivity disorder, autism spectrum disorder, learning difficulties, and intellectual disability compared to the general population [12,22,33,56,62,75,90,91,92,93,94,95,96,97,98,99]. It is well-known that attention-deficit/hyperactivity disorder is more prevalent in children with congenital heart defects than in healthy controls. The studies conducted to date mainly use parent-report screening methods or include in their assessment only certain categories of children with congenital heart defects considered to be at high risk, resulting in a variable prevalence of attention-deficit/hyperactivity disorder between 5% and 44% [56,62,75,90,91,92,93,94]. Studies using parent rating scales of attention-deficit/hyperactivity symptoms have shown rates of attention problems in the clinically significant range for 17.8% of children with hypoplastic left heart syndrome, 30% of those with complex congenital heart malformations, and 44% of children following neonatal aortic arch repair [75,92,93]. In a study conducted by Loblein et al. on a population of 206 children with congenital heart defects aged between 3 and 21 years, in which neuropsychological assessment was performed by a licensed psychologist, the rate of attention-deficit/hyperactivity disorder was 27% compared to the general population (9.04%) [61]. In studies using more rigorous diagnostic methods, the rate of attention-deficit/hyperkinetic disorders is higher in certain groups of children with congenital heart defects (34% in adolescents with single-ventricle congenital heart disease versus 6%; 24% in adolescents with tetralogy of Fallot versus 5%; 16% in adolescents with transposition of the great arteries versus 3%) [56,62,95]. This highlights the need for more rigorous diagnostic methods (neuropsychological and neurodevelopmental assessment) and regular evaluations rather than screening in order to identify autism and attention disorders as early as possible in children with congenital heart defects. Autism spectrum disorder is common in children with congenital heart defects. In a study conducted by Razzaghi et al., children aged 2–17 with congenital heart defects were more likely to have autism spectrum disorder than children without congenital heart defects [90]. In a case–control study conducted by Sigmon et al., patients with congenital heart defects showed an increased probability of autism diagnosis [96]. In a study conducted by Tan et al. on a group of 134 children with congenital heart defects, 5.9% of them were diagnosed with autism during clinical evaluation. Children at risk for autism showed significant delays in adaptive functioning, such as the ability to engage in simple activities, poor social functioning, and low communication skills. Therefore, repeated assessments throughout development are necessary to improve the quality of diagnosis. In the case of two patients in the study with suspected autism, the symptoms were not reported by the parents at the time of presentation for evaluation [97]. Repeated screening and long-term follow-up by a specialized professional of this at-risk population is necessary regardless of parental concerns. Screening should also be conducted using diagnostic methods with high sensitivity and specificity to identify and provide early intervention for patients with congenital heart defects who are at increased risk for autism spectrum disorder. In the future, it would be useful to analyze modifiable risk factors such as age at diagnosis, the timing and quality of intervention, and the types of services provided. Early diagnosis of autism and intervention can change the course of outcomes for children and their families. This presents a major opportunity for all practitioners working with children with congenital heart defects to increase their awareness and respective training, either to incorporate screening/assessment as part of routine care or to refer the patient to a specialist.

In a study conducted by Gonzales et al., using objective data (diagnosis codes and medication records), it was shown that adolescents with any type of congenital heart defect have a higher prevalence of anxiety and/or depression compared to their peers without congenital heart defects (19–23% vs. 6–9%) [94]. By using objective diagnostic methods instead of self-report surveys to assess anxiety and depression, the prevalence data obtained can be generalizable to a broader population of children and adolescents with congenital heart defects. Adolescents with congenital heart malformations due to stressful events and multiple surgical procedures may experience acute stress reactions, post-traumatic stress reactions (12–14%), or even post-traumatic stress disorder in 12–31% of cardiac surgery groups, which leads to psychological and behavioral impairment and increased use of healthcare services. In this context, early screening for psychosocial problems is necessary, and referral for psychosocial treatment (trauma-focused cognitive behavioral therapy) should be considered when indicated [14].

### 4.3. Neurodevelopmental Outcomes

Children with congenital heart defects are at increased risk for neurodevelopmental problems that impact their ability to reach typical developmental milestones [96,99,100,101]. In a study conducted by Delgado et al., it was shown that children with congenital heart defects are 15 times more likely to have intellectual disabilities and approximately 13 times more likely to have other health impairments compared to children without congenital heart defects [27]. Children with severe congenital heart defects are at higher risk for developmental delays, compared to those with less severe congenital heart defects [27,101,102]. The presence of additional risk factors, such as a genetic disorder, increases the risk of global developmental delay [61]. Also, the presence of specific medical factors contributes to the appearance of specific neuropsychological disorders (e.g., impaired executive function) in children with congenital heart defects, even if the patient does not meet the criteria for a neurodevelopmental disorder [61]. For example, patients with congenital two-ventricle heart disease, aortic obstruction and prematurity show impaired executive function [89,103,104]. Children with congenital heart malformations with aortic obstruction may be at increased risk for neurodevelopmental disorders, likely due to the impact on prenatal cerebral blood flow with consequences for fetal brain development. Studies have shown that children with a small aortic diameter at birth exhibit differences in white matter development in deep brain regions, and these types of abnormalities have been associated with neuropsychological and neurodevelopmental outcomes [105,106]. Children with congenital heart defects are at increased risk for learning disorders that affect their educational progress. In a study conducted by Wright et al., which compared a group of 29 children with Fallot’s tetralogy or transposition of the great arteries, surgically corrected, with a group of children with heart murmurs that did not require treatment, significantly lower scores were found in the domains of reading, spelling, and arithmetic [107]. It is important to note that the neurodevelopmental profile changes with age. For example, preschool children tend to show global developmental delays. Attention disorders, impaired executive function and cognitive impairment involved in the learning process may occur with school integration. Given the developmental trajectory and the fact that attention-deficit, hyperkinetic disorder, or specific learning disorders cannot be diagnosed in early childhood, these children may “grow into” certain deficits that may arise during development [108]. This highlights the importance of continuous monitoring throughout development, even if initial assessments are normal, with a focus on measures that can best predict neuro neurocognitive (e.g., screening for executive function, assessment of phonological skills). Children with congenital heart defects should be monitored for neurodevelopmental and neurocognitive disorders in both childhood and adolescence [61]. Early identification of neurodevelopmental disorders followed by specific intervention is associated with greater academic gains and improved outcomes in children with developmental disabilities [109].

### 4.4. Cognitive Outcomes

Adolescents with congenital heart defects must cope with both age-specific challenges (self-development, independence, autonomy, and social relationships) and stressors related to their illness [110]. In the context of congenital heart defects, typical adolescent development can be affected by a number of factors, such as: impaired body image due to postoperative scarring, absence from school, frequent hospitalizations, physical limitations and parental overprotection, which can lead to psychosocial problems [37]. Studies have shown that adolescents with congenital heart defects who have undergone a large number of surgical procedures, lower systemic saturation and complex heart defects are at significantly increased risk for cognitive problems, including intelligence, executive functioning, attention, and academic performance, compared with their peers [56,62]. Impaired executive function in children with congenital heart defects may have long-term functional consequences. With a better understanding of the specific patterns of cognitive vulnerabilities experienced by children with congenital heart defects, it is possible to identify risks early on and provide individualized support to achieve the best possible outcomes.

### 4.5. Psychosocial Functioning and Quality of Life

Congenital heart diseases in children and adolescents are associated with a negative psychosocial impact and impaired quality of life [73]. Compared to healthy children of the same age, patients with congenital heart defects have a lower quality of life and this can be exacerbated by the severity of the condition and the complexity of the surgical intervention [111,112,113]. Quantifying quality of life, which generally includes physical, mental and social well-being, provides information on treatment effectiveness and healthcare quality. It can provide useful information in clinical practice by identifying risk factors that could impair patient outcomes [73]. There is therefore an urgent need for healthcare professionals to promote psychosocial well-being among children and adolescents with congenital heart defects, in order to improve their quality of life. However, understanding the psychological impact of congenital heart defects is an important step towards creating evidence-based, patient-oriented interventions [70]. Once mental health comorbidities are identified, it is important to increase resilience in order to improve the quality of life of these patients, as demonstrated in pediatric cancer patients or adolescents with diabetes [114,115]. Studies have shown correlations between increased resilience and depression in adolescents with congenital heart defects [116]. In a pilot study conducted by Cousino et al. on a group of 20 adolescents with Fontan palliated CHD, the feasibility, acceptability and effectiveness of a new group-based telemedicine psycho-education program were demonstrated [24]. However, it is necessary to study the effects of the intervention in the future across multiple centers, among various populations, and using different implementation methods.

Children and adolescents with congenital heart defects are at increased risk for psychological issues, psychiatric disorders and neurodevelopmental disabilities with reduced quality of life, especially those with complex congenital heart defects. In this context, prevention, early diagnosis and a multidisciplinary approach are essential. It is also necessary to identify modifiable medical risk factors and establish concrete strategies to improve long-term functional prognosis [117].

Psychological outcomes in children with CHD are likely influenced not only by medical factors but also by psychosocial and environmental variables. Family context, parental mental health, socioeconomic status, and access to psychological support may all play significant roles in shaping developmental trajectories and emotional adjustment. This highlights the importance of adopting a biopsychosocial perspective when interpreting the findings.

Although the available intervention studies suggest promising directions for improving psychological outcomes in children with CHD, the current evidence base remains limited. Most studies are characterized by small sample sizes, heterogeneity in intervention design, and a lack of randomized controlled trials. Therefore, these findings should be interpreted with caution, as they primarily indicate feasibility and potential benefit rather than established efficacy. Future research should prioritize well-designed, adequately powered studies to better define the effectiveness of psychological and behavioral interventions in this population.

### 4.6. Strengths and Limitations

#### 4.6.1. Strengths

An important strength of this review is the structured differentiation between psychiatric, neurodevelopmental, cognitive and psychosocial outcomes, which are often examined separately in the literature but rarely synthesized within a unified framework.

#### 4.6.2. Limitations of the Evidence

The findings of this review must be interpreted in light of several limitations inherent to the available literature. First, substantial clinical and methodological heterogeneity was observed across studies, including differences in CHD severity classification, age at assessment, outcome measures and follow-up duration. Differences in assessment tools may partly explain inconsistencies across studies and highlight the need for greater standardization in future research.

Differences in informant perspectives should also be considered when interpreting the findings, as discrepancies between parent reports and self-reports are well-documented in pediatric psychological research.

Each patient must be assessed individually to achieve the best results. The use of certain tools, such as scales, questionnaires, and surveys, has limited utility and does not always yield accurate results due to a lack of standardization, subjectivity, and imprecise scoring systems—especially in psychiatry, where the presence and intensity of symptoms are difficult to measure objectively. Long-term follow-up of patients is necessary to determine the true prevalence of psychological issues in patients with congenital heart defects. Second, many studies relied on cross-sectional designs, restricting causal inference and limiting understanding of developmental trajectories. Third, population-based registry studies often lacked detailed clinical and psychosocial variables, whereas clinic-based samples may have overrepresented more severe cases, potentially introducing selection bias. Finally, intervention studies were few, frequently underpowered and heterogeneous in design, reducing the certainty of conclusions regarding effectiveness.

The generalizability of the findings may be limited, as many studies were conducted in specialized tertiary care centers. These populations may differ from broader CHD populations in terms of disease severity, access to care, and follow-up, which may influence psychological outcomes.

#### 4.6.3. Limitations of the Review Process

This review also has methodological limitations. Although conducted according to PRISMA 2020 guidelines, the protocol was not prospectively registered in a public database. Only English-language publications were included, which may have introduced language bias. Due to the high heterogeneity of study designs and outcomes, no meta-analysis was performed, and findings were synthesized narratively.

The overall risk of bias was predominantly moderate, largely driven by the observational nature of the evidence, potential confounding factors, and variability in outcome assessment methods. Smaller cross-sectional studies were particularly susceptible to selection bias and limited generalizability.

The overall certainty of evidence was limited by the predominance of observational designs and methodological heterogeneity, which should be considered when interpreting the findings.

Additionally, unpublished studies and gray literature were not systematically searched.

### 4.7. Implications for Practice and Future Research

The findings of this review have important clinical implications. Routine psychological and neurodevelopmental screening should be integrated into the long-term care of children with CHD, particularly for those with complex conditions.

A multidisciplinary approach, including cardiology, psychology, and developmental specialists, is essential to address the diverse needs of this population. Early identification of vulnerabilities may facilitate timely intervention and improve long-term outcomes.

Future research should aim to improve consistency in outcome measurement and clearly differentiate between developmental stages. Longitudinal studies are particularly needed to better understand the evolution of psychological outcomes from early childhood into adolescence. Additionally, larger and methodologically robust studies, including controlled intervention trials, are required to strengthen the evidence base and improve comparability across studies.

### 4.8. Key Messages

Overall, three main conclusions can be drawn from this review. First, children and adolescents with CHD are at increased risk for neurodevelopmental and cognitive impairments, particularly in the context of complex disease. Second, psychiatric and psychosocial outcomes are more variable and influenced by both medical and environmental factors. Third, psychological outcomes evolve across developmental stages, highlighting the need for age-specific assessment and intervention strategies.

## 5. Conclusions

In conclusion, children and adolescents with congenital heart defects are at increased risk for a range of neurodevelopmental, cognitive, and psychosocial challenges. These outcomes are influenced by both medical and environmental factors and evolve across development. Future research should focus on methodological consistency and longitudinal approaches to better inform clinical care.

## Figures and Tables

**Table 1:** Summary of main psychological, neurodevelopmental and psychosocial outcomes in children and adolescents with congenital heart defects, including key findings, representative studies and clinical implications.

Outcome Domain	Developmental Stage	Main Findings	Association with Severity of CHD	Key Studies	Clinical Implications
Psychiatric	Early childhood/School age/Adolescence	↑ ADHD, ASD ↑ anxiety, depression	Variable	Tsao 2017 [22] Miles 2023 [16] Liu 2024 [20] Jaworski 2017 [10] Konkel et al. 2023 [18] Azim et al. 2021 [23]	Mental health screening and targeted psychological interventions
Neurodevelopment	Infancy/Early childhood	Delays in motor, language, and global development; distinct developmental trajectories; higher risk in complex CHD	Strong	Cainelli et al. 2021 [21] Davidson et al. 2015 [25]	Early developmental screening and intervention programs are essential
Cognitive outcomes	School age	Lower IQ scores, executive dysfunction, academic difficulties	Moderate–Strong	Tsao 2017 [22] Liu 2024 [20]	Neuropsychological evaluation and educational support strategies
Psychosocial and Quality of Life	Adolescence	Reduced HRQoL, social difficulties, strong influence of parental well-being; potential resilience factors	Weak–Moderate	Raj 2019 [28] Grimaldi Capitello 2025 [19] Cousino 2025 [24]	Psychological support Family-centered care and resilience-building interventions

Abbreviations: CHD = congenital heart defects; ADHD = attention-deficit/hyperactivity disorder; ASD = autism spectrum disorder; HRQoL = health-related quality of life. ↑ means increased.

## Data Availability

No new data were created or analyzed in this study. Data sharing is not applicable to this article.

## Supplementary Materials

The following supporting information can be downloaded at: https://www.mdpi.com/article/10.3390/diagnostics16091271/s1, Supplementary File S1: PRISMA checklist; Supplementary File S2: Search strategy; Supplementary File S3: Characteristics of included studies; Supplementary File S4: Studies that appeared eligible but were excluded after full-text review; Supplementary File S5: Risk of bias assessment summary.

## Abbreviations

The following abbreviations are used in this manuscript:

CHD	Congenital Heart Defect
ASD	Autism Spectrum Disorder
ADHD	Attention-Deficit/Hyperactivity Disorder
PTSD	Post-Traumatic Stress Disorder
PTSR	Post-Traumatic Stress Reaction
RR	Risk Ratio
OD	Odds Ratio
HR	Hazard Ratio
MD	Mean Difference
SMD	Standardized Mean Difference

## References

[1] Liu Y., Chen S., Zühlke L., Black G.C., Choy M.K., Li N., Keavney B.D. (2019). Global birth prevalence of congenital heart defects 1970–2017: Updated systematic review and meta-analysis of 260 studies. Int. J. Epidemiol..

[2] Kovacs A.H., Brouillette J., Ibeziako P., Jackson J.L., Kasparian N.A., Kim Y.Y., Livecchi T., Sillman C., Kochilas L.K., American Heart Association Council on Lifelong Congenital Heart Disease and Heart Health in the Young (2022). Psychological Outcomes and Interventions for Individuals with Congenital Heart Disease: A Scientific Statement From the American Heart Association. Circ. Cardiovasc. Qual. Outcomes.

[3] Gilboa S.M., Devine O.J., Kucik J.E., Oster M.E., Riehle-Colarusso T., Nembhard W.N., Xu P., Correa A., Jenkins K., Marelli A.J. (2016). Congenital Heart Defects in the United States: Estimating the Magnitude of the Affected Population in 2010. Circulation.

[4] Carazo M.R., Kolodziej M.S., DeWitt E.S., Kasparian N.A., Newburger J.W., Duarte V.E., Singh M.N., Opotowsky A.R. (2020). Prevalence and Prognostic Association of a Clinical Diagnosis of Depression in Adult Congenital Heart Disease: Results of the Boston Adult Congenital Heart Disease Biobank. J. Am. Heart Assoc..

[5] Benderly M., Kalter-Leibovici O., Weitzman D., Blieden L., Buber J., Dadashev A., Mazor-Dray E., Lorber A., Nir A., Yalonetsky S. (2019). Depression and anxiety are associated with high health care utilization and mortality among adults with congenital heart disease. Int. J. Cardiol..

[6] Stout K.K., Daniels C.J., Aboulhosn J.A., Bozkurt B., Broberg C.S., Colman J.M., Crumb S.R., Dearani J.A., Fuller S., Gurvitz M. (2019). 2018 AHA/ACC Guideline for the Management of Adults With Congenital Heart Disease: Executive Summary: A Report of the American College of Cardiology/American Heart Association Task Force on Clinical Practice Guidelines. J. Am. Coll. Cardiol..

[7] Liu H.-C., Chaou C.-H., Lo C.-W., Chung H.-T., Hwang M.-S. (2022). Factors Affecting Psychological and Health-Related Quality-of-Life Status in Children and Adolescents with Congenital Heart Diseases. Children.

[8] Marino B.S., Lipkin P.H., Newburger J.W., Peacock G., Gerdes M., Gaynor J.W., Mussatto K.A., Uzark K., Goldberg C.S., Johnson W.H. (2012). Neurodevelopmental outcomes in children with congenital heart disease: Evaluation and management: A scientific statement from the American Heart Association. Circulation.

[9] Marelli A., Miller S.P., Marino B.S., Jefferson A.L., Newburger J.W. (2016). Brain in Congenital Heart Disease Across the Lifespan: The Cumulative Burden of Injury. Circulation.

[10] Jaworski J.L.B., Flynn T., Burnham N., Chittams J.L., Sammarco T., Gerdes M., Bernbaum J.C., Clancy R.R., Solot C.B., Zackai E.H. (2017). Rates of autism and potential risk factors in children with congenital heart defects. Congenit. Heart Dis..

[11] Yamada D.C., Porter A.A., Conway J.L., LeBlanc J.C., Shea S.E., Hancock-Friesen C.L., Warren A.E. (2013). Early repair of congenital heart disease associated with increased rate of attention deficit hyperactivity disorder symptoms. Can. J. Cardiol..

[12] Hansen E., Poole T.A., Nguyen V., Lerner M., Wigal T., Shannon K., Wigal S.B., Batra A.S. (2012). Prevalence of ADHD symptoms in patients with congenital heart disease. Pediatr. Int..

[13] Marshall K.H., D’Udekem Y., Sholler G.F., Opotowsky A.R., Costa D.S.J., Sharpe L., Celermajer D.S., Winlaw D.S., Newburger J.W., Kasparian N.A. (2020). Health-Related Quality of Life in Children, Adolescents, and Adults With a Fontan Circulation: A Meta-Analysis. J. Am. Heart Assoc..

[14] Meentken M.G., van Beynum I.M., Legerstee J.S., Helbing W.A., Utens E.M. (2017). Medically Related Post-traumatic Stress in Children and Adolescents with Congenital Heart Defects. Front. Pediatr..

[15] Luyckx K., Goossens E., Rassart J., Apers S., Vanhalst J., Moons P. (2014). Parental support, internalizing symptoms, perceived health status, and quality of life in adolescents with congenital heart disease: Influences and reciprocal effects. J. Behav. Med..

[16] Miles K.G., Farkas D.K., Laugesen K., Sørensen H.T., Kasparian N.A., Madsen N. (2023). Mental Health Conditions Among Children and Adolescents With Congenital Heart Disease: A Danish Population-Based Cohort Study. Circulation.

[17] Page M.J., McKenzie J.E., Bossuyt P.M., Boutron I., Hoffmann T.C., Mulrow C.D., Shamseer L., Tetzlaff J.M., Akl E.A., Brennan S.E. (2021). The PRISMA 2020 statement: An updated guideline for reporting systematic reviews. BMJ.

[18] Konkel T., Kroll K.H., Goertz M.T., Lavoie J., Bagli S.P., Kogutkiewicz K., Kostroski R., Scott L., Stoll P., Andres J. (2023). Screening for traumatic stress in children and adolescents with congenital heart disease. Prog. Pediatr. Cardiol..

[19] Grimaldi Capitello T., Correale C., Amodeo G., Balsamo M., Carlucci L., Fiorilli C. (2025). Childhood heart disease and parental emotional wellbeing: A predictive model to explain the perception of quality of life in children and adolescents. Health Qual. Life Outcomes.

[20] Liu Z.Y., Wang Q.Q., Pang X.Y., Huang X.B., Yang G.M., Zhao S. (2024). Association of congenital heart disease and neurodevelopmental disorders: An observational and Mendelian randomization study. Ital. J. Pediatr..

[21] Cainelli E., Bisiacchi P.S., Cogo P., Padalino M., Simonato M., Vergine M., Lanera C., Vedovelli L. (2021). Detecting neurodevelopmental trajectories in congenital heart diseases with a machine-learning approach. Sci. Rep..

[22] Tsao P.C., Lee Y.S., Jeng M.J., Hsu J.W., Huang K.L., Tsai S.J., Chen M.H., Soong W.J., Kou Y.R. (2017). Additive effect of congenital heart disease and early developmental disorders on attention-deficit/hyperactivity disorder and autism spectrum disorder: A nationwide population-based longitudinal study. Eur. Child Adolesc. Psychiatry.

[23] Azim K., Sheikh A.M., Khokhar M.M., Kanwal A., Akbar T., Masood S. (2021). Anxiety and depression in children and adolescents with congenital heart disease before and after surgical intervention period. Pak. Armed Forces Med. J..

[24] Cousino M.K., Rea K.E., Dusing C.R., Glenn T., Armstrong B., Yu S., Lowery R., Les A.S., Goldberg C.S., Hansen J.E. (2025). A pilot study of the WE BEAT Well-Being Education Programme to build resilience in adolescents with heart disease. Cardiol. Young.

[25] Davidson J., Gringras P., Fairhurst C., Simpson J. (2015). Physical and neurodevelopmental outcomes in children with single-ventricle circulation. Arch. Dis. Child..

[26] Ernst M.M., Marino B.S., Cassedy A., Piazza-Waggoner C., Franklin R.C., Brown K., Wray J. (2018). Biopsychosocial Predictors of Quality of Life Outcomes in Pediatric Congenital Heart Disease. Pediatr. Cardiol..

[27] Delgado C., Ullery M.A., Zeng G., Simpson E.A., Tanner J.P., Kirby R.S., Duclos C., Lowry J., Salemi J.L. (2023). Elevated risk for developmental disabilities in children with congenital heart defects. Birth Defects Res..

[28] Raj M., Sudhakar A., Roy R., Champaneri B., Sudevan R., Kabali C., Kumar R.K. (2019). Health-related quality of life (HRQOL) in children and adolescents with congenital heart disease: A cross-sectional survey from South India. BMJ Paediatr. Open.

[29] Schmithorst V.J., Panigrahy A., Gaynor J.W., Watson C.G., Lee V., Bellinger D.C., Rivkin M.J., Newburger J.W. (2016). Organizational topology of brain and its relationship to ADHD in adolescents with D-transposition of the great arteries. Brain Behav..

[30] Pike N.A., Roy B., Moye S., Cabrera-Mino C., Woo M.A., Halnon N.J., Lewis A.B., Kumar R. (2021). Reduced hippocampal volumes and memory deficits in adolescents with single ventricle heart disease. Brain Behav..

[31] Eichler A., Köhler-Jonas N., Stonawski V., Purbojo A., Moll G.H., Heinrich H., Cesnjevar R.A., Kratz O. (2019). Child neurodevelopment and mental health after surgical ventricular septal defect repair: Risk and protective factors. Dev. Med. Child Neurol..

[32] Dulfer K., Duppen N., Van Dijk A.P., Kuipers I.M., Van Domburg R.T., Verhulst F.C., Van der Ende J., Helbing W.A., Utens E.M. (2015). Parental mental health moderates the efficacy of exercise training on health-related quality of life in adolescents with congenital heart disease. Pediatr. Cardiol..

[33] El Sehmawy A.A., Younes Abd Elaziz S., Abdelghany Elsheikh A., Elsawy F.A., Abd Elsalam Amin A., Mostafa Omran A., Younan Abd El Malek A. (2024). Assessment of mental health and quality of life among children with congenital heart disease. J. Pediatr. Rehabil. Med..

[34] Ramanan S., Sundaram S., Gopalakrishnan A., Anija D.V., Sandhya P., Jose D.S., Baruah S.D., Menon S., Dharan B.S. (2021). Intermediate-term neurodevelopmental outcomes and quality of life after arterial switch operation beyond early neonatal period. Eur. J. Cardio-Thorac. Surg..

[35] Ramanan S., Gopalakrishnan A., Sundaram S., Varma R.P., Gopakumar D., Viswam V.K., Satheesan R., Baruah S.D., Menon S., Dharan B.S. (2023). Paediatric quality of life in toddlers and children who underwent arterial switch operation beyond early neonatal period. Eur. J. Cardiothorac. Surg..

[36] Sarrechia I., Miatton M., De Wolf D., François K., Gewillig M., Meyns B., Vingerhoets G. (2016). Neurocognitive development and behaviour in school-aged children after surgery for univentricular or biventricular congenital heart disease. Eur. J. Cardiothorac. Surg..

[37] Moon J.R., Song J., Huh J., Kang I.-S., Park S.W., Chang S.-A., Yang J.-H., Jun T.-G. (2017). The Relationship between Parental Rearing Behavior, Resilience, and Depressive Symptoms in Adolescents with Congenital Heart Disease. Front. Cardiovasc. Med..

[38] Chen H., Yan Y., Li C., Zheng X., Wang G., Jin Z., Shi G., He X., Tong X., Chen H. (2022). Inattention and hyperactivity in children and adolescents with repaired D-transposition of the great arteries: Prevalence, perioperative risk factors, and clinical outcomes. Front. Cardiovasc. Med..

[39] Seivert N.P., Dodds K.M., Demianczyk A., Goldberg D.J., Rychik J. (2025). Promoting emotional & behavioral health for pediatric patients with Fontan circulation: Integrating psychology into a dedicated multidisciplinary clinic. Front. Cardiovasc. Med..

[40] Wehrle F.M., Furrer M., Feldmann M., Liamlahi R., Naef N., O’gOrman R., Latal B., Huber R. (2023). Functional networks of working memory abilities in children with complex congenital heart disease: A sleep EEG study. Child Neuropsychol..

[41] Maya S., Gunawijaya E., Yantie N.P.V.K., Windiani I.G.A.T. (2020). Growth, Development, and Quality of Life in Children with Congenital Heart Disease. Open Access Maced. J. Med. Sci..

[42] Milo F., Calcagni G., Maiolo S., Drago F., Vicari S., Capitello T.G., Menghini D., Rossi A. (2024). Health-related quality of life among paediatric patients with coarctation of the aorta: An observational study. Psychol. Health Med..

[43] Jassal Y.R., Kelly S., DiMaria M., Jacobsen R., Brigham D., Hawkins S., Rafferty C., Wolfe K.R. (2023). Implications of attention and executive functioning weaknesses in youth with Fontan circulation. Child Neuropsychol..

[44] Wang C.C., Weng W.C., Chang L.Y., Chang H.-Y., Wu M.-H., Wang J.-K., Lu C.-W., Lin M.-T., Chen C.-A., Chiu S.-N. (2021). Increased prevalence of inattention-related symptoms in a large cohort of patients with congenital heart disease. Eur. Child Adolesc. Psychiatry.

[45] Noori N., Teimouri A., Boryri T., Shafiee S.S. (2017). Quality of Life in Children and Adolescents with Congenital Heart Diseases in Zahedan, Iran. J. Pediatr. Perspect..

[46] Lepage C., Tremblay L., Bernier A., Simard M.N., Matte-Gagné C., Gallagher A. (2025). Trajectories of behavioral and emotional problems in preschoolers with congenital heart disease. J. Pediatr. Psychol..

[47] Schmitt K.R.L., Sievers L.K., Hütter A., Abdul-Khaliq H., Poryo M., Berger F., Bauer U.M.M., Helm P.C., Pfitzer C. (2023). New Insights into the Education of Children with Congenital Heart Disease with and without Trisomy 21. Medicina.

[48] Zampi J.D., Heinrich K.P., Bergersen L., Goldstein B.H., Batlivala S.P., Fuller S., Glatz A.C., O’bYrne M.L., Marino B., Afton K. (2024). Neurocognitive function and health-related quality of life in adolescents and young adults with CHD with pulmonary valve dysfunction. Cardiol. Young.

[49] Pike N.A., Roy B., Gupta R., Singh S., Woo M.A., Halnon N.J., Lewis A.B., Kumar R. (2018). Brain abnormalities in cognition, anxiety, and depression regulatory regions in adolescents with single ventricle heart disease. J. Neurosci. Res..

[50] McWhorter L.G., Christofferson J., Neely T., Hildenbrand A.K., Alderfer M.A., Randall A., Kazak A.E., Sood E. (2022). Parental post-traumatic stress, overprotective parenting, and emotional and behavioural problems for children with critical congenital heart disease. Cardiol. Young.

[51] Noorani S., Roy B., Sahib A.K., Cabrera-Mino C., Halnon N.J., Woo M.A., Lewis A.B., Pike N.A., Kumar R. (2020). Caudate nuclei volume alterations and cognition and mood dysfunctions in adolescents with single ventricle heart disease. J. Neurosci. Res..

[52] Khanna A.D., Duca L.M., Kay J.D., Shore J., Kelly S.L., Crume T. (2019). Prevalence of Mental Illness in Adolescents and Adults With Congenital Heart Disease from the Colorado Congenital Heart Defect Surveillance System. Am. J. Cardiol..

[53] Neal A.E., Stopp C., Wypij D., Bellinger D.C., Dunbar-Masterson C., DeMaso D.R., Newburger J.W. (2015). Predictors of health-related quality of life in adolescents with tetralogy of Fallot. J. Pediatr..

[54] Mulkey S.B., Bai S., Luo C., Cleavenger J.E., Gibson N., Holland G., Mosley B.S., Kaiser J.R., Bhutta A.T. (2016). School-Age Test Proficiency and Special Education After Congenital Heart Disease Surgery in Infancy. J. Pediatr..

[55] Calderon J., Stopp C., Wypij D., DeMaso D.R., Rivkin M., Newburger J.W., Bellinger D.C. (2016). Early-Term Birth in Single-Ventricle Congenital Heart Disease After the Fontan Procedure: Neurodevelopmental and Psychiatric Outcomes. J. Pediatr..

[56] Holland J.E., Cassidy A.R., Stopp C., White M.T., Bellinger D.C., Rivkin M.J., Newburger J.W., DeMaso D.R. (2017). Psychiatric Disorders and Function in Adolescents with Tetralogy of Fallot. J. Pediatr..

[57] Calderon J., Wypij D., Rofeberg V., Stopp C., Roseman A., Albers D., Newburger J.W., Bellinger D.C. (2020). Randomized Controlled Trial of Working Memory Intervention in Congenital Heart Disease. J. Pediatr..

[58] Cassedy A., Wray J., Qadir A.A., Ernst M.M., Brown K., Franklin R., Wernovsky G., Marino B.S. (2023). Behavioral and Emotional Outcomes in Children with Congenital Heart Disease: Effects of Disease Severity, Family Life Stress, Disease-Related Chronic Stress, and Psychosocial Adaptation. J. Pediatr..

[59] Zampi J.D., Ilardi D.L., McCracken C.E., Yun Z., Glatz A.C., Goldstei B.H., Petit C.J., Qureshi A.M., Goldberg C.S., Law M.A. (2025). Comparing Parent Perception of Neurodevelopment after Primary versus Staged Repair of Neonatal Symptomatic Tetralogy of Fallot. J. Pediatr..

[60] Rassart J., Luyckx K., Goossens E., Oris L., Apers S., Moons P., on behalf of the i-DETACH Investigators (2016). A Big Five Personality Typology in Adolescents with Congenital Heart Disease: Prospective Associations with Psychosocial Functioning and Perceived Health. Int. J. Behav. Med..

[61] Loblein H.J., Vukmirovich P.W., Donofrio M.T., Sanz J.H. (2023). Prevalence of neurodevelopmental disorders in a clinically referred sample of children with CHD. Cardiol. Young.

[62] DeMaso D.R., Calderon J., Taylor G.A., Holland J.E., Stopp C., White M.T., Bellinger D.C., Rivkin M.J., Wypij D., Newburger J.W. (2017). Psychiatric Disorders in Adolescents With Single Ventricle Congenital Heart Disease. Pediatrics.

[63] Eckerström F., Hjortdal V.E., Rask C.U., Nyboe C. (2024). Psychiatric morbidity and work participation in patients with congenital ventricular septal defects: A case-controlled study. Eur. Heart J. Qual. Care Clin. Outcomes.

[64] Sakshi S., Ramakrishnan S. (2025). Quality of life in children with congenital heart disease: An emerging Indian perspective. Ann. Pediatr. Cardiol..

[65] Grosch I.B., Andresen B., Diep L.M., Diseth T.H., Möller T. (2022). Quality of life and emotional vulnerability in a national cohort of adolescents living with Fontan circulation. Cardiol. Young.

[66] Seivert N.P., Dodds K.M., O’Malley S., Goldberg D.J., Paridon S., McBride M., Rychik J. (2025). Associations Between Exercise Capacity and Psychological Functioning in Children and Adolescents with Fontan Circulation. Pediatr. Cardiol..

[67] Bircan E., Politis M.D., Gokun Y., Luo C., Leonard H., Bourke J., Bower C., Nembhard W.N. (2023). Intellectual disabilities and autism among children with congenital heart defects, Western Australia, 1983–2010. BMC Pediatr..

[68] Czobor N.R., Ocsovszky Z., Roth G., Takács S., Csabai M., Székely E., Gál J., Székely A., Thege B.K. (2021). ADHD symptomatology of children with congenital heart disease 10 years after cardiac surgery: The role of age at operation. BMC Psychiatry.

[69] Ehrler M., Bellinger D.C., Cassidy A.R., Newburger J.W., Calderon J. (2023). Social cognition and behavioral outcomes in congenital heart disease: Profiles and neuropsychiatric comorbidities. Child Neuropsychol..

[70] So S.C.Y., Li W.H.C., Ho K.Y. (2019). The impact of congenital heart disease on the psychological well-being and quality of life of Hong Kong Chinese adolescents: A cross-sectional study. J. Clin. Nurs..

[71] Sertçelik T., Alkan F., Sapmaz Ş.Y., Coşkun Ş., Eser E. (2018). Life quality of children with congenital heart diseases. Turk. Pediatri Ars..

[72] Lee J.S., Blais A., Jackson J., Patel B.J., Lai L., Goldfield G., Sananes R., Longmuir P.E. (2021). Higher Child-Reported Internalizing and Parent-Reported Externalizing Behaviors were Associated with Decreased Quality of Life among Pediatric Cardiac Patients Independent of Diagnosis: A Cross-Sectional Mixed-Methods Assessment. Congenit. Heart Dis..

[73] Xiang L., Su Z., Liu Y., Huang Y., Zhang X., Li S., Zhang H. (2019). Impact of Family Socioeconomic Status on Health-Related Quality of Life in Children With Critical Congenital Heart Disease. J. Am. Heart Assoc..

[74] Dalziel K.M., Hulse E.S.G., Huang L., Marshall K.H., Cordina R., Du Plessis K., Sholler G., Celermajer D., Winlaw D., D’ACoz Y.D. (2025). Health-related quality-of-life for people with a Fontan circulation, their parents, and siblings. Eur. J. Cardiovasc. Nurs..

[75] Sistino J.J., Atz A.M., Simpson K.N., Ellis C., Ikonomidis J.S., Bradley S.M. (2015). The prevalence of attention-deficit/hyperactivity disorder following neonatal aortic arch repair. Cardiol. Young.

[76] Feldmann M., Ullrich C., Bataillard C., Knirsch W., Gosteli-Peter M.A., Latal B., Held U. (2019). Neurocognitive outcome of school-aged children with congenital heart disease who underwent cardiopulmonary bypass surgery: A systematic review protocol. Syst. Rev..

[77] Duijff S.N., Klaassen P.W., de Veye H.F., Beemer F.A., Sinnema G., Vorstman J.A. (2012). Cognitive development in children with 22q11.2 deletion syndrome. Br. J. Psychiatry J. Ment. Sci..

[78] Roos-Hesselink J.W., Meijboom F.J., Spitaels S.E., Van Domburg R., Van Rijen E.H., Utens E.M., Bogers A.J., Simoons M.L. (2004). Outcome of patients after surgical closure of ventricular septal defect at young age: Longitudinal follow-up of 22–34 years. Eur. Heart J..

[79] Spector L.G., Menk J.S., Knight J.H., McCracken C., Thomas A.S., Vinocur J.M., Oster M.E., St Louis J.D., Moller J.H., Kochilas L. (2018). Trends in Long-Term Mortality After Congenital Heart Surgery. J. Am. Coll. Cardiol..

[80] Nagaraj U.D., Evangelou I.E., Donofrio M.T., Vezina L.G., McCarter R., du Plessis A.J., Limperopoulos C. (2015). Impaired Global and Regional Cerebral Perfusion in Newborns with Complex Congenital Heart Disease. J. Pediatr..

[81] Coe D.A., Matson J.L., Russell D.W., Slifer K.J., Capone G.T., Baglio C., Stallings S. (1999). Behavior problems of children with Down syndrome and life events. J. Autism Dev. Disord..

[82] Lisanti A.J. (2018). Parental stress and resilience in CHD: A new frontier for health disparities research. Cardiol. Young.

[83] Chiperi L.E., Hutanu A. (2024). Glial Fibrillary Acidic Protein: Diagnostic and Prognostic Role in Psychomotor Development Dynamics in Patients with Congenital Heart Defects after Cardiovascular Surgery. Balk. Med. J..

[84] Kovacs A.H., Luyckx K., Thomet C., Budts W., Enomoto J., Sluman M.A., Lu C.W., Jackson J.L., Khairy P., Cook S.C. (2024). Anxiety and Depression in Adults With Congenital Heart Disease. J. Am. Coll. Cardiol..

[85] Chen W., Chen H., Jiang W., Chen C., Xu M., Ruan H., Chen H., Yu Z., Chen S. (2025). Heart rate variability and heart rate asymmetry in adolescents with major depressive disorder during nocturnal sleep period. BMC Psychiatry.

[86] Ali F., Ladak L.A., Usmani A., Javaid M., Anwar T., Hasan B.S. (2025). Health-Related Quality of Life, Challenges, and Experiences of Patients With Congenital Heart Disease After Cardiac Catheterization: A Mixed-Methods Study. J. Soc. Cardiovasc. Angiogr. Interv..

[87] Sadhwani A., Cheng H., Stopp C., Rollins C.K., Jolley M.A., Dunbar-Masterson C., Wypij D., Newburger J., Ware J., Thiagarajan R.R. (2019). Early Neurodevelopmental Outcomes in Children Supported with ECMO for Cardiac Indications. Pediatr. Cardiol..

[88] Sadhwani A., Butler S., Rofeberg V., Espinosa K., Wood L., Cassidy A.R., Calderon J., Rollins C.K., Singer J., Henson B. (2023). Sleep Patterns in Young Children with Congenital Heart Disease. J. Pediatr..

[89] Gerstle M., Beebe D.W., Drotar D., Cassedy A., Marino B.S. (2016). Executive Functioning and School Performance among Pediatric Survivors of Complex Congenital Heart Disease. J. Pediatr..

[90] Razzaghi H., Oster M., Reefhuis J. (2015). Long-term outcomes in children with congenital heart disease: National Health Interview Survey. J. Pediatr..

[91] Holst L.M., Kronborg J.B., Jepsen J.R.M., Christensen J.Ø., Vejlstrup N.G., Juul K., Bjerre J.V., Bilenberg N., Ravn H.B. (2020). Attention-deficit/hyperactivity disorder symptoms in children with surgically corrected Ventricular Septal Defect, Transposition of the Great Arteries, and Tetralogy of Fallot. Cardiol. Young.

[92] Mahle W.T., Clancy R.R., Moss E.M., Gerdes M., Jobes D.R., Wernovsky G. (2000). Neurodevelopmental outcome and lifestyle assessment in school-aged and adolescent children with hypoplastic left heart syndrome. Pediatrics.

[93] Shillingford A.J., Glanzman M.M., Ittenbach R.F., Clancy R.R., Gaynor J.W., Wernovsky G. (2008). Inattention, hyperac tivity, and school performance in a population of school-age children with complex Congenital Heart Disease. Pediatrics.

[94] Gonzalez V.J., Kimbro R.T., Cutitta K.E., Shabosky J.C., Bilal M.F., Penny D.J., Lopez K.N. (2021). Mental Health Disorders in Children With Congenital Heart Disease. Pediatrics.

[95] DeMaso D.R., Labella M., Taylor G.A., Forbes P.W., Stopp C., Bellinger D.C., Rivkin M.J., Wypij D., Newburger J.W. (2014). Psychiatric disorders and function in adolescents with d-transposition of the great arteries. J. Pediatr..

[96] Sigmon E.R., Kelleman M., Susi A., Nylund C.M., Oster M.E. (2019). Congenital Heart Disease and Autism: A Case-Control Study. Pediatrics.

[97] Tan A., Semmel E.S., Wolf I., Hammett B., Ilardi D. (2020). Implementing standard screening for autism spectrum disorder in CHD. Cardiol. Young.

[98] Huisenga D., La Bastide-Van Gemert S., Van Bergen A., Sweeney J., Hadders-Algra M. (2021). Developmental outcomes after early surgery for complex congenital heart disease: A systematic review and meta-analysis. Dev. Med. Child Neurol..

[99] Feldmann M., Bataillard C., Ehrler M., Ullrich C., Knirsch W., Gosteli-Peter M.A., Held U., Latal B. (2021). Cognitive and executive function in congenital heart disease: A meta-analysis. Pediatrics.

[100] Khalil A., Suff N., Thilaganathan B., Hurrell A., Cooper D., Carvalho J.S. (2014). Brain abnormalities and neurodevelopmental delay in congenital heart disease: Systematic review and meta-analysis. Ultrasound Obstet. Gynecol..

[101] Oster M.E., Watkins S., Hill K.D., Knight J.H., Meyer R.E. (2017). Academic Outcomes in Children With Congenital Heart Defects: A Population-Based Cohort Study. Circ. Cardiovasc. Qual. Outcomes.

[102] Riehle-Colarusso T., Autry A., Razzaghi H., Boyle C.A., Mahle W.T., Van Naarden Braun K., Correa A. (2015). Congenital Heart Defects and Receipt of Special Education Services. Pediatrics.

[103] Sanz J.H., Berl M.M., Armour A.C., Wang J., Cheng Y.I., Donofrio M.T. (2017). Prevalence and pattern of executive dysfunction in school age children with congenital heart disease. Congenit. Heart Dis..

[104] Sanz J.H., Wang J., Berl M.M., Armour A.C., Cheng Y.I., Donofrio M.T. (2018). Executive Function and Psychosocial Quality of Life in School Age Children with Congenital Heart Disease. J. Pediatr..

[105] Zaidi A.H., Newburger J.W., Wypij D., Stopp C., Watson C.G., Friedman K.G., Rivkin M.J., Rollins C.K. (2018). Ascending Aorta Size at Birth Predicts White Matter Microstructure in Adolescents Who Underwent Fontan Palliation. J. Am. Heart Assoc..

[106] Fleming M., Athanasopoulos P., Mackay D.F., Pell J.P. (2024). Educational outcomes among children with congenital heart disease compared to peers: A Scotland-wide record-linkage study of 715,850 schoolchildren. BMC Pediatr..

[107] Wright M., Nolan T. (1994). Impact of cyanotic heart disease on school performance. Arch. Dis. Child..

[108] Brosig C.L., Bear L., Allen S., Simpson P., Zhang L., Frommelt M., Mussatto K.A. (2018). Neurodevelopmental outcomes at 2 and 4 years in children with congenital heart disease. Congenit. Heart Dis..

[109] Glotzbach K.L., Ward J.J., Marietta J., Eckhauser A.W., Winter S., Puchalski M.D., Miller T.A. (2020). The Benefits and Bias in Neurodevelopmental Evaluation for Children with Congenital Heart Disease. Pediatr. Cardiol..

[110] Jackson J.L., Gerardo G.M., Monti J.D., Schofield K.A., Vannatta K. (2018). Executive Function and Internalizing Symptoms in Adolescents and Young Adults With Congenital Heart Disease: The Role of Coping. J. Pediatr. Psychol..

[111] Mellion K., Uzark K., Cassedy A., Drotar D., Wernovsky G., Newburger J.W., Mahony L., Mussatto K., Cohen M., Limbers C. (2014). Health-related quality of life outcomes in children and adolescents with congenital heart disease. J. Pediatr..

[112] Ong L.C., Teh C.S., Darshinee J., Omar A., Ang H.L. (2017). Quality of life of Malaysian children with CHD. Cardiol. Young.

[113] McCrindle B.W., Williams R.V., Mitchell P.D., Hsu D.T., Paridon S.M., Atz A.M., Li J.S., Newburger J.W., Pediatric Heart Network Investigators (2006). Relationship of patient and medical characteristics to health status in children and adolescents after the Fontan procedure. Circulation.

[114] Rosenberg A.R., Salsman J.M. (2024). Resilience in adolescent and young adult oncology: Problems and prospects. Cancer.

[115] Yi-Frazier J.P., Yaptangco M., Semana S., Buscaino E., Thompson V., Cochrane K., Tabile M., Alving E., Rosenberg A.R. (2015). The association of personal resilience with stress, coping, and diabetes outcomes in adolescents with type 1 diabetes: Variable- and person-focused approaches. J. Health Psychol..

[116] Moon J.R., Huh J., Kang I.-S., Park S.W., June T.-G., Lee H.J. (2006). Relationship between depression and resilience in adolescents with congenital heart disease. Am. Heart Assoc..

[117] Wernovsky G., Licht D.J. (2016). Neurodevelopmental Outcomes in Children With Congenital Heart Disease—What Can We Impact?. Pediatr. Crit. Care Med..

