# Supplementary material for: Psychological Aspects and Mental Health Risks in Children and Adolescents with Congenital Heart Defects—A Systematic Review
Source: Diagnostics (Basel). 2026 Apr 23;16(9):1271. doi: 10.3390/diagnostics16091271 (PMC13163961; doi:10.3390/diagnostics16091271)
Supplement: Supplementary file 1 [file diagnostics-16-01271-s001.zip › Supplementary file S2 Search strategy.pdf]

Supplementary file S2 - Search strategy

PubMed

("Heart Defects, Congenital"[Mesh]

OR "congenital heart disease"[tiab]

OR "congenital heart defect*"[tiab])

AND

("Child"[Mesh] OR "Adolescent"[Mesh]

OR child*[tiab] OR adolescen*[tiab]

OR pediatric*[tiab] OR paediatric*[tiab])

AND

("Depressive Disorder"[Mesh]

OR "Anxiety Disorders"[Mesh]

OR "Autism Spectrum Disorder"[Mesh]

OR "Attention Deficit Disorder with Hyperactivity"[Mesh]

OR "Stress Disorders, Post-Traumatic"[Mesh]

OR depression[tiab]

OR anxiety[tiab]

OR ADHD[tiab]

OR autism[tiab]

OR PTSD[tiab]

OR "psychiatric disorder*"[tiab]

OR "mental disorder*"[tiab]

OR "behavior problem*"[tiab]

OR "emotional problem*"[tiab])

Embase (Elsevier)

('congenital heart disease'/exp

OR 'congenital heart disease':ti,ab

OR 'congenital heart defect*':ti,ab)

AND

('child'/exp OR 'adolescent'/exp

OR child*:ti,ab OR adolescen*:ti,ab

OR pediatric*:ti,ab OR paediatric*:ti,ab)

AND

('depression'/exp

OR 'anxiety disorder'/exp

OR 'autism spectrum disorder'/exp

OR 'attention deficit disorder'/exp

OR 'posttraumatic stress disorder'/exp

OR depression:ti,ab

OR anxiety:ti,ab

OR ADHD:ti,ab

OR autism:ti,ab

OR PTSD:ti,ab

OR 'psychiatric disorder*':ti,ab

OR 'mental disorder*':ti,ab

OR 'behavior problem*':ti,ab

OR 'emotional problem*':ti,ab)

Scopus (Elsevier)

(TITLE-ABS-KEY("congenital heart disease" OR "congenital heart defect*" OR "heart defect*, congenital"))

AND

(TITLE-ABS-KEY(child* OR adolescen* OR pediatric* OR paediatric*))

AND

(TITLE-ABS-KEY(depression OR anxiety OR ADHD OR autism OR PTSD

OR "psychiatric disorder*" OR "mental disorder*"

OR "behavior problem*" OR "emotional problem*"))

Web of Science (Clarivate)

TS=("congenital heart disease" OR "congenital heart defect*")

AND

TS=(child* OR adolescen* OR pediatric* OR paediatric*)

AND

TS=(depression OR anxiety OR ADHD OR autism OR PTSD

OR "psychiatric disorder*" OR "mental disorder*"

OR "behavior problem*" OR "emotional problem*")

Cochrane Library

("congenital heart disease" OR "congenital heart defect*")

AND

(child* OR adolescen* OR pediatric* OR paediatric*)

AND

(depression OR anxiety OR ADHD OR autism OR PTSD

OR "psychiatric disorder*" OR "mental disorder*"

OR "behavior problem*" OR "emotional problem*")
