# Supplementary material for: Psychological Aspects and Mental Health Risks in Children and Adolescents with Congenital Heart Defects—A Systematic Review
Source: Diagnostics (Basel). 2026 Apr 23;16(9):1271. doi: 10.3390/diagnostics16091271 (PMC13163961; doi:10.3390/diagnostics16091271)
Supplement: Supplementary file 1 [file diagnostics-16-01271-s001.zip › Supplementary file S3 Characteristics of included studies.pdf]

Supplementary file S3. Characteristics of included studies

Authors (Year), Country	Study design	Sample size & participants	Outcomes assessed	Instruments / methods	Parental / family variables	Main results & effect estimates	Predictors / moderators	Key findings
Konkel et al. (2023), Germany	Cross-sectional	~180 children/adolescents with CHD; mixed severity	PTSD / traumatic stress	UCLA PTSD Index, screening tools	Parental distress (reported)	~20–25% screened positive for traumatic stress	Disease complexity, prior surgery	Clinically relevant traumatic stress common; supports routine screening
Grimaldi Capitello et al. (2025), Italy	Cross-sectional, SEM	300+ children/adolescents with CHD + parents	QoL, emotional wellbeing	PedsQL, parental wellbeing scales	Parental emotional wellbeing	β paths significant (p<0.01)	Parental emotional status	Parent wellbeing strongly predicts child QoL perception
Liu et al. (2024), China	Observational + Mendelian randomization	>10,000 registry cases	Neurodevelopmental disorders	Registry data, genetic instruments	–	OR ~1.3–1.6 for NDD	Genetic liability	Evidence for causal CHD–NDD link
Cainelli et al. (2021), Italy	Cohort + ML	140 infants/children with CHD	Neurodevelopmental trajectories	Bayley scales, ML clustering	–	Distinct developmental clusters identified	CHD complexity	ML identifies vulnerable neurodevelopmental subgroups
Bean Jaworski et al. (2017), USA	Cohort	~200 children with CHD	ASD prevalence	ADOS, DSM criteria	–	ASD prevalence ~7–10%	Genetic syndromes	ASD rates higher than population
Tsao et al. (2017), Taiwan	Population-based cohort	>17,000 CHD + controls	ADHD, ASD	National registry	–	HR ~1.5–2.0	Early developmental disorders	Additive risk of CHD and EDD

Authors (Year), Country	Study design	Sample size & participants	Outcomes assessed	Instruments / methods	Parental / family variables	Main results & effect estimates	Predictors / moderators	Key findings
Azim et al. (2021), Pakistan	Pre–post surgical	120 children/adolescents	Anxiety, depression	HADS / CDI	–	Scores ↓ post-surgery ($p<0.05$)	Surgery	Psychological symptoms improve after repair
Cousino et al. (2025), USA	Pilot intervention	30 adolescents with CHD	Resilience, wellbeing	WE BEAT program	Family support	Moderate effect sizes	Program participation	Feasible resilience intervention
Davidson et al. (2015), UK	Cohort	60 children with single ventricle	Neurodevelopment	Standardized developmental tests	–	Lower scores vs norms	Single ventricle physiology	Persistent neurodevelopmental risk
Ernst et al. (2018), UK/USA	Cross-sectional	250 children/adolescents	QoL	PedsQL	Family stress	β significant for psychosocial factors	Psychosocial stress	Biopsychosocial model explains QoL
Delgado et al. (2023), USA	Population cohort	>25,000 CHD	Developmental disability	Birth defects registry	–	RR ~1.8	CHD severity	Elevated DD risk at population level
Raj et al. (2019), India	Cross-sectional	500 children/adolescents	HRQoL	PedsQL	–	Lower QoL vs norms	Disease severity	QoL impaired across domains
Schmithorst et al. (2016), USA	Neuroimaging	49 adolescents with D-TGA	ADHD traits	fMRI network analysis	–	Altered topology vs controls	Brain organization	Neural correlates of ADHD
Pike et al. (2021), USA	MRI study	30 adolescents, single ventricle	Memory	MRI volumetry, memory tests	–	↓ hippocampal volume; d~0.8	Single ventricle	Structural–cognitive link
Liu H-C et al. (2022), Taiwan	Cross-sectional	180 children/adolescents	QoL, mental health	PedsQL, SDQ	Family support	Significant correlations	Family support	Psychosocial factors shape QoL

Authors (Year), Country	Study design	Sample size & participants	Outcomes assessed	Instruments / methods	Parental / family variables	Main results & effect estimates	Predictors / moderators	Key findings
Eichler et al. (2019), Germany	Cohort	90 post-VSD repair	Neurodevelopment	IQ, CBCL	Parental education	Small–moderate deficits	SES, parental factors	Mostly good outcomes with subtle risks
Dulfer et al. (2015), Netherlands	RCT (exercise)	93 adolescents	QoL	PedsQL	Parental mental health	Interaction effect ($p<0.05$)	Parental mental health	Parent MH moderates intervention efficacy
El Sehmawy et al. (2024), Egypt	Cross-sectional	150 children	Anxiety, depression, QoL	CDI, SCAS, PedsQL	–	Higher symptom burden	Disease severity	Mental health impairment common
Ramanan et al. (2021), India	Cohort	120 post-ASO	Neurodevelopment, QoL	Bayley, PedsQL	–	Persistent mild deficits	Age at surgery	Later ASO linked to worse outcomes
Ramanan et al. (2023), India	Cohort	200 toddlers/children	QoL	PedsQL	–	Lower physical/social scores	Surgery timing	QoL affected beyond infancy
Sarrechia et al. (2016), Belgium	Cohort	125 school-age	Cognition, behavior	WISC, CBCL	–	Lower IQ/executive scores	Uni- vs biventricular	Univentricular worse outcomes
Moon et al. (2017), Korea	Cross-sectional	134 adolescents	Depression	CES-D	Parenting style	$r \approx 0.3–0.4$	Rearing behavior	Parenting influences depression
Chen et al. (2022), China	Cohort	80 D-TGA	ADHD symptoms	Conners scales	–	ADHD prevalence ↑	Perioperative factors	Surgery-related predictors
Seivert et al. (2025), USA	Program description	Fontan clinic cohort	Emotional/behavioral health	Integrated psychology model	Family engagement	Descriptive improvement	Multidisciplinary care	Embedded psychology beneficial

Authors (Year), Country	Study design	Sample size & participants	Outcomes assessed	Instruments / methods	Parental / family variables	Main results & effect estimates	Predictors / moderators	Key findings
Wehrle et al. (2023), Switzerland	EEG study	35 complex CHD	Working memory	Sleep EEG	–	Altered networks	CHD complexity	Neurophysiological correlates
Maya et al. (2020), Indonesia	Cross-sectional	100 children	Growth, development, QoL	Developmental screening	–	Delays common	Nutrition, severity	Multidimensional impact
Milo et al. (2024), Italy	Observational	90 coarctation	HRQoL	PedsQL	–	Mildly reduced QoL	Residual obstruction	Even "simple" CHD affected
Jassal et al. (2023), USA	Neuropsychological	70 Fontan	Executive function	Neuropsych tests	–	Large deficits (d>0.8)	Fontan physiology	Executive dysfunction prominent
Wang et al. (2021), Taiwan	Population cohort	>3,000 CHD	Inattention	ADHD rating scales	–	OR ~1.4	Cyanotic CHD	Inattention prevalent
Noori et al. (2017), Iran	Cross-sectional	160 children	QoL	PedsQL	–	Lower QoL vs controls	Disease severity	QoL impairment
Lepage et al. (2025), Canada	Longitudinal	150 preschoolers	Behavior trajectories	CBCL	Parenting stress	Distinct trajectories	Early stress	Early behavioral vulnerability
Schmitt et al. (2023), Germany	Cross-sectional	200 school-age	Education outcomes	School records	Family SES	Lower attainment	Trisomy 21	Educational needs high
Miles et al. (2023), Denmark	Population cohort	>15,000 CHD	Psychiatric diagnoses	National registry	–	HR ~1.6	Age, severity	Elevated MH burden
Zampi et al. (2024), USA	Cross-sectional	110 adolescents/YA	Cognition, QoL	Neuropsych tests	–	Moderate deficits	Valve dysfunction	Late sequelae evident
Pike et al. (2018), USA	MRI study	25 single ventricle	Anxiety, depression	MRI + scales	–	Structural differences	Limbic regions	Brain–emotion links

Authors (Year), Country	Study design	Sample size & participants	Outcomes assessed	Instruments / methods	Parental / family variables	Main results & effect estimates	Predictors / moderators	Key findings
McWhorter et al. (2022), USA	Cohort	120 families	Child behavior	CBCL	Parental PTSD	Significant associations	Parental PTSD	Parent trauma affects child outcomes
Noorani et al. (2020), USA	MRI study	28 adolescents	Cognition, mood	MRI volumetry	–	Caudate volume ↓	Single ventricle	Subcortical involvement
Khanna et al. (2019), USA	Registry	>9,000	Mental illness	ICD codes	–	Prevalence ↑	Age	Lifelong MH risk
Neal et al. (2015), USA	Cohort	130 ToF	QoL	PedsQL	–	Predictors identified	Exercise capacity	Functional status matters
Mulkey et al. (2016), USA	Cohort	250 school-age	Academic achievement	School tests	Parental education	Higher special ed use	Infant surgery	Educational impact
Calderon et al. (2016), USA	Cohort	150 Fontan	Neurodevelopment, psych	IQ, DSM	–	Early-term birth risk	Gestational age	Perinatal modifiers
Holland et al. (2017), USA	Cohort	125 ToF	Psychiatric disorders	Structured interview	–	↑ anxiety/depression	Disease severity	Psychiatric morbidity common
Calderon et al. (2020), USA	RCT	80 children	Working memory	Cogmed	Family support	Small–moderate gains	Intervention	Cognitive training beneficial
Cassedy et al. (2023), USA	Cross-sectional	300 children	Behavior, emotion	CBCL	Family stress	β significant	Family stress	Psychosocial load key
Jeffrey et al. (2025), USA	Cohort	90 neonatal ToF	Neurodevelopment	Parent report	–	No major differences	Repair strategy	Parent perception varies
Rassart et al. (2016), Belgium	Longitudinal	135 adolescents	Personality, psychosocial	Big Five	Parental support	Prospective associations	Personality	Personality profiles matter
Loblein et al. (2023), USA	Clinical cohort	200 referrals	NDD prevalence	Clinical assessment	–	High prevalence	Referral bias	Referred samples high risk

Authors (Year), Country	Study design	Sample size & participants	Outcomes assessed	Instruments / methods	Parental / family variables	Main results & effect estimates	Predictors / moderators	Key findings
DeMaso et al. (2017), USA	Cohort	156 single ventricle	Psychiatric disorders	DSM interview	–	~30% disorder prevalence	SV physiology	High psychiatric burden
Eckerström et al. (2024), Denmark	Case–control	400 VSD	Psychiatric morbidity	Registry	–	OR ~1.2–1.4	SES	Even VSD linked to MH risk
Sakshi & Ramakrishnan (2025), India	Narrative / cross-sectional	Pediatric CHD	QoL	PedsQL	–	Consistently reduced	Contextual factors	Emerging LMIC data
Grosch et al. (2022), Norway	National cohort	170 Fontan	QoL, emotion	PedsQL	–	Lower QoL	Disease burden	Emotional vulnerability
Seivert et al. (2025), USA	Cohort	90 Fontan	Psych functioning	Standard scales	Family activity	r significant	Exercise capacity	Physical–psych link
Bircan et al. (2023), Australia	Population cohort	1,200 CHD	ID, ASD	Registry linkage	–	RR ~1.7	Genetic factors	Long-term NDD risk
Czobor et al. (2021), Hungary	Cohort	100 post-surgery	ADHD symptoms	ADHD scales	–	Age-at-op effect	Surgery age	Later surgery protective
Ehrler et al. (2023), USA	Neuropsych	140 children	Social cognition	Theory of Mind tasks	–	Distinct profiles	Comorbidity	Social cognition affected
So et al. (2019), Hong Kong	Cross-sectional	180 adolescents	Wellbeing, QoL	PedsQL	–	Lower emotional QoL	Severity	Cultural consistency
Sertçelik et al. (2018), Turkey	Cross-sectional	120 children	QoL	PedsQL	–	Reduced QoL	Disease severity	Universal QoL impact

Authors (Year), Country	Study design	Sample size & participants	Outcomes assessed	Instruments / methods	Parental / family variables	Main results & effect estimates	Predictors / moderators	Key findings
Lee et al. (2021), Canada	Mixed-methods	90 children	Internalizing/externalizing	CBCL, interviews	Parent report	Associations with QoL	Behavior	Behavior predicts QoL
Xiang et al. (2019), China	Cohort	300 critical CHD	QoL	PedsQL	Family SES	SES gradient	Socioeconomic status	Social determinants crucial
Dalziel et al. (2025), Europe	Cross-sectional	Fontan families	QoL (child, parent, sibs)	EQ-5D, PedsQL	Family-wide	Lower family QoL	Disease burden	CHD affects entire family
Sistino et al. (2015), USA	Cohort	110 neonates	ADHD prevalence	DSM diagnosis	–	ADHD ~15–20%	Arch repair	Early surgery risk
