# Supplementary material for: Psychological Aspects and Mental Health Risks in Children and Adolescents with Congenital Heart Defects—A Systematic Review
Source: Diagnostics (Basel). 2026 Apr 23;16(9):1271. doi: 10.3390/diagnostics16091271 (PMC13163961; doi:10.3390/diagnostics16091271)
Supplement: Supplementary file 1 [file diagnostics-16-01271-s001.zip › Supplementary file S4 Studies that appeared eligible but were excluded after full-text review.pdf]

Supplementary file S4. Studies that appeared eligible but were excluded after full-text review

Study (Author, Year)	Title (Shortened)	Reason for Exclusion
**Feldmann et al., 2019**	Neurocognitive effects of cardiopulmonary bypass in children with CHD	Only a systematic review protocol, no data available
**Duijff et al., 2012**	Cognitive development in patients with 22q11.2 deletion syndrome	Focused exclusively on a genetic syndrome; psychological outcomes not linked specifically to CHD.
**Roos-Hesselinket al., 2004**	Mortality and surgical outcomes after VSD repair	Assessed only cardiac/surgical endpoints; no mental health or neurodevelopmental outcomes.
**Spector et al., 2018**	Long-term mortality after cardiac surgery in mixed-age CHD cohorts	Mixed pediatric–adult sample without extractable data for participants ≤18 years.
**Nagaraj et al., 2015**	Postoperative cerebral perfusion in neonates with complex CHD	Examined only intraoperative neurophysiology; lacked psychological or cognitive assessments.
**Coe et al., 1999**	Behavioral functioning in children with Down syndrome and CHD	Outcomes attributed to the genetic condition; CHD effects not isolated; excluded per criteria.
**Lisanti et al., 2018**	Parental stress after infant cardiac surgery	Outcomes measured exclusively in parents; no child psychological or developmental data.
**Chiperi et al., 2024**	biomarkers after cardiovascular surgery	Focused on biomarker; no neurodevelopmental or psychological follow-up.
**Kovacs et al., 2024**	Anxiety and coping in adults operated for CHD	Adult-only population (>18 years).

Study (Author, Year)	Title (Shortened)	Reason for Exclusion
**Chen, 2025**	Heart rate variability and emotional regulation in adolescents with depression	No CHD population included
**Ali et al., 2025**	Quality of life after catheterization for CHD	no neurodevelopmental or psychological data
**Sadhwani et al., 2019**	Neurodevelopment after early-life ECMO in CHD	Severe heterogeneity: combined CHD and non-CHD sample; pediatric CHD-specific results not extractable.
**Sadhwani et al., 2023**	Sleep patterns in hospitalized infants with CHD	Only physiological measures of sleep; no psychological or behavioral outcomes.
**Gerstle et al., 2016**	Executive function in CHD survivors	neurodevelopmental outcomes not reported; study population included children and their parents
