# Supplementary material for: Psychological Aspects and Mental Health Risks in Children and Adolescents with Congenital Heart Defects—A Systematic Review
Source: Diagnostics (Basel). 2026 Apr 23;16(9):1271. doi: 10.3390/diagnostics16091271 (PMC13163961; doi:10.3390/diagnostics16091271)
Supplement: Supplementary file 1 [file diagnostics-16-01271-s001.zip › Supplementary file S5 Risk of Bias Assessment Summary.pdf]

Supplementary File S5. Risk of Bias Assessment Summary

**Study, year**	**Type of study**	**Instrument**	**Overall risk of bias**	**Key Issues**
Dulfer et al. (2015)	RCT	**RoB 2.0**	Some concerns	Blinding unclear, allocation concealment not detailed
Calderon et al. (2020)	RCT	**RoB 2.0**	Some concerns	Missing data + unclear outcome assessor blinding
Azim et al. (2021)	Non-randomized interventional	**ROBINS-I**	Serious	No control group, pre–post design
Cousino et al. (2025)	Non-randomized interventional	**ROBINS-I**	Serious	Pilot, small sample, selection bias
Seivert et al. (2025)	Non-randomized interventional	**ROBINS-I**	Serious	Descriptive program, no comparator
Tsao et al. (2017)	Large population-based / registry study	**ROBINS-I**	Low	Well-adjusted registry
Miles et al. (2023)	Large population-based / registry study	**ROBINS-I**	Low	Large cohort, strong methodology
Khanna et al. (2019)	Large population-based / registry study	**ROBINS-I**	Moderate	Registry limitations

**Study, year**	**Type of study**	**Instrument**	**Overall risk of bias**	**Key Issues**
Delgado et al. (2023)	Large population-based / registry study	**ROBINS-I**	Low	Large population-based
Liu et al. (2024)	Large population-based / registry study	**ROBINS-I**	Moderate	MR assumptions
Wang et al. (2021)	Large population-based / registry study	**ROBINS-I**	Moderate	Residual confounding
Cainelli et al. (2021)	Cohort study	**ROBINS-I**	Moderate	ML exploratory
Bean Jaworski et al. (2017)	Cohort study	**ROBINS-I**	Moderate	Confounding (genetics)
Davidson et al. (2015)	Cohort study	**ROBINS-I**	Moderate	Small sample
Eichler et al. (2019)	Cohort study	**ROBINS-I**	Moderate	SES confounding
Ramanan et al. (2021)	Cohort study	**ROBINS-I**	Moderate	Timing bias
Ramanan et al. (2023)	Cohort study	**ROBINS-I**	Moderate	No control group
Sarrechia et al. (2016)	Cohort study	**ROBINS-I**	Moderate	Sample size
Lepage et al. (2025)	Cohort study	**ROBINS-I**	Moderate	Behavioral trajectories subjective

**Study, year**	**Type of study**	**Instrument**	**Overall risk of bias**	**Key Issues**
Schmitt et al. (2023)	Cohort study	**ROBINS-I**	Moderate	Educational confounders
McWhorter et al. (2022)	Cohort study	**ROBINS-I**	Moderate	Parental PTSD self-report
Neal et al. (2015)	Cohort study	**ROBINS-I**	Moderate	Functional confounding
Mulkey et al. (2016)	Cohort study	**ROBINS-I**	Moderate	Education bias
Calderon et al. (2016)	Cohort study	**ROBINS-I**	Moderate	Perinatal confounders
Holland et al. (2017)	Cohort study	**ROBINS-I**	Moderate	Psychiatric assessment variability
Konkel et al. (2023)	Cross-sectional study	**ROBINS-I**	Moderate	Self-report PTSD
Grimaldi Capitello et al. (2025)	Cross-sectional study	**ROBINS-I**	Moderate	SEM model assumptions
Ernst et al. (2018)	Cross-sectional study	**ROBINS-I**	Moderate	Psychosocial confounding
Raj et al. (2019)	Cross-sectional study	**ROBINS-I**	Moderate	No longitudinal data
Liu H-C et al. (2022)	Cross-sectional study	**ROBINS-I**	Moderate	Family factors
El Sehmawy et al. (2024)	Cross-sectional study	**ROBINS-I**	Moderate	Measurement bias
Moon et al. (2017)	Cross-sectional study	**ROBINS-I**	Moderate	Parenting self-report

Study, year	Type of study	Instrument	Overall risk of bias	Key Issues
Maya et al. (2020)	Cross-sectional study	**ROBINS-I**	Moderate	Screening tools only
Milo et al. (2024)	Cross-sectional study	**ROBINS-I**	Moderate	Mild disease heterogeneity
Noori et al. (2017)	Cross-sectional study	**ROBINS-I**	Moderate	QoL subjective
Zampi et al. (2024)	Cross-sectional study	**ROBINS-I**	Moderate	Cross-sectional cognition
Jassal et al. (2023)	Cross-sectional study	**ROBINS-I**	Moderate	Neuropsych variability
Chen et al. (2022)	Cross-sectional study	**ROBINS-I**	Serious	Small + perioperative bias
Wehrle et al. (2023)	Cross-sectional study	**ROBINS-I**	Serious	EEG small sample
Pike et al. (2021)	Cross-sectional study	**ROBINS-I**	Serious	MRI small sample
Pike et al. (2018)	Cross-sectional study	**ROBINS-I**	Serious	Neuroimaging small cohort
Noorani et al. (2020)	Cross-sectional study	**ROBINS-I**	Serious	Very small MRI sample
Schmithorst et al. (2016)	Neuroimaging study	**ROBINS-I**	Serious	fMRI exploratory
Pike et al. (2021)	Neuroimaging study	**ROBINS-I**	Serious	n<50
Noorani et al. (2020)	Neuroimaging study	**ROBINS-I**	Serious	volumetric limits

Study, year	Type of study	Instrument	Overall risk of bias	Key Issues
Wehrle et al. (2023)	Neuroimaging study	**ROBINS-I**	Serious	EEG small sample

Abbreviations: RoB 2.0, Revised Cochrane Risk of Bias tool; ROBINS-I, Risk Of Bias In Non-randomized Studies of Interventions; ML, Machine Learning; MR, Mendelian Randomization; SEM, Structural Equation Modeling; SES, socioeconomic status; PTSD, post-traumatic stress disorder; QoL, quality of life; EEG, electroencephalography; MRI, magnetic resonance imaging.
